# Developmental Toxicity of Ibrutinib: Insights from Stem Cell Dynamics and Neural Regeneration in Planarians

**DOI:** 10.3390/biom15121665

**Published:** 2025-11-29

**Authors:** Weiyun Guo, Baijie Jin, Nannan Li, Dandan Sun, Dezeng Liu, Zimei Dong, Guangwen Chen

**Affiliations:** 1 College of Life Science, Henan Normal University, Xinxiang 453007, China; 1904084041@stu.htu.edu.cn (W.G.); jinbaijie@htu.edu.cn (B.J.); 2104283129@stu.htu.edu.cn (N.L.); 1904283091@stu.htu.edu.cn (D.S.); 2022027@htu.edu.cn (D.L.); 2 School of Life Sciences and Technology, Henan Medical University, Xinxiang 453003, China

**Keywords:** ibrutinib, stem cells, planarian, toxicity, *BTK*

## Abstract

Ibrutinib (IB), a Bruton’s tyrosine kinase (BTK) inhibitor, is widely used against B-cell malignancies. However, its adverse effects on stem cell-dependent processes and tissue homeostasis remain incompletely understood. Freshwater planarians possess pluripotent stem cells (neoblasts), which enable remarkable regeneration of various tissues, including the central nervous system. This makes them ideal in vivo models for studying chemical toxicity within a whole-organism context. Here, we utilized planarian *Dugesia constrictiva* to assess IB toxicity and elucidate its mechanisms, focusing on its impact on stem cell dynamics and regeneration. Our results demonstrated that exposure to IB at concentrations as low as 0.9 mg/L, far below clinical plasma levels, led to severe morphological and regenerative impairments, including disrupted neural regeneration. Mechanistically, IB disrupted stem cell dynamics by suppressing proliferation and differentiation and by inducing oxidative stress via ROS overproduction. Notably, IB exposure significantly downregulated *BTK* expression. Crucially, *BTK* RNAi caused the key toxic effects of IB exposure, including morphological and regenerative defects, suppression of stem cell proliferation and differentiation, and increased apoptosis. Therefore, we conclude that IB may exert its toxicity in planarians primarily through *BTK* inhibition. This finding provides direct functional evidence linking *BTK* inhibition to stem cell dysfunction and regenerative defects in a novel in vivo context, offering critical insights for refining the clinical safety profile of IB.

## 1. Introduction

Bruton’s tyrosine kinase (BTK), a member of the Tec kinase family, is a critical regulator of B-cell receptor signaling pathways, which govern B-cell development, proliferation, and differentiation [1]. The dysregulation of BTK activity has been strongly associated with hematological malignancies and autoimmune disorders. Emerging evidence indicates that BTK signaling plays critical roles in embryonic morphogenesis and neurogenesis [2], making BTK inhibitors promising candidates for targeted therapies. Ibrutinib (IB), the first-in-class BTK inhibitor approved by the FDA in 2013, exerts its effect by covalently binding to BTK, thereby effectively disrupting downstream B-cell survival signaling cascades [3]. It has emerged as a highly effective therapeutic agent for various B-cell malignancies, including chronic lymphocytic leukemia (CLL), mantle cell lymphoma (MCL), small lymphocytic lymphoma, and primary central nervous system lymphoma [4,5,6].

Despite its therapeutic effectiveness, growing evidence raises concerns about the potential developmental toxicity of IB. Although IB has received FDA approval for pediatric chronic graft-versus-host disease (GVHD) [7,8], the existing clinical data are insufficient for a comprehensive safety evaluation in the pediatric population. The pivotal iMAGINE trial (NCT03790332) excluded infants from its study cohort and primarily focused on short-term efficacy rather than long-term developmental consequences, thereby limiting a comprehensive assessment of IB’s potential impact on child growth and development. Furthermore, early mammalian toxicology studies conducted by the FDA in 2017 revealed that IB exhibited dose-dependent embryotoxicity at 2–20 times the clinical exposure level in rats, leading to fetal growth restriction, visceral malformations (particularly cardiovascular defects), and significantly increased embryo lethality [9]. Rabbit models further confirmed its teratogenicity and fetal resorption, prompting the classification of IB as a confirmed developmental toxicant. A study by Wang et al. using zebrafish demonstrated that IB exposure induces dose-dependent teratogenicity, such as neural tube defects, organ maldevelopment, and neurobehavioral abnormalities, suggesting the evolutionary conservation of its developmental toxicity [10]. These findings underscore the urgent need for systematic investigations into the developmental toxicity mechanisms of IB. However, the molecular mechanisms of IB-induced developmental toxicity remain unclear. In particular, whether its developmental toxicity is mediated through the impairment of stem cell function requires further in-depth elucidation. Moreover, traditional mammalian models exhibit significant limitations in evaluating these effects, highlighting the critical need to establish more sensitive and efficient novel model systems.

The freshwater planarian, an invertebrate model known for its extraordinary regenerative abilities, provides a unique platform to tackle these scientific challenges. Planarians are endowed with abundant pluripotent stem cells, referred to as neoblasts, which constitute about 30% of their total somatic cells and are evenly distributed throughout the organism [11,12]. Following injury, neoblasts precisely reconstruct all tissues, including the central nervous system (CNS), within seven days through tightly regulated proliferation and differentiation programs [13,14]. Remarkably, this systemic, stem cell-driven regeneration closely resembles key processes of mammalian embryonic development, providing a dynamic in vivo system to study chemical disruption of morphogenesis. Furthermore, planarians were thought to be one of the most primitive organisms to possess anterior cephalization (“brains”) and were ostensibly represented as an ancestor of the human brain [13,15]. Additional advantages include experimental tractability, well-defined anatomy, and heightened sensitivity to toxicants. Most importantly, unlike conventional in vitro systems, the planarian model preserves the native stem cell niche within a whole-organism context, delivering physiologically relevant insights into drug-induced stem cell and developmental toxicity [13].

In this study, we utilize the freshwater planarian *Dugesia constrictiva* to systematically track the toxic effects of IB exposure across individual, cellular, and molecular levels during regeneration. We selected this species due to its regional availability, which allows for the maintenance of a genetically standardized colony. Additionally, its established sensitivity, which yields clear, quantifiable responses to chemical exposure [16], makes it ideal for reproducible toxicological assessment. By elucidating the effects of IB on stem cell dynamics and neural regeneration, and characterizing the underlying molecular mechanisms, our findings will provide crucial mechanistic insights into the developmental toxicity of IB. These findings will help inform safer clinical use in pediatric populations and contribute to improved monitoring strategies for developmental disorders associated with BTK inhibitor therapy.

## 2. Materials and Methods

### 2.1. Animals and Treatment

A laboratory-bred clonal strain of *D. constrictiva* was maintained in autoclaved tap water at 20 °C under constant darkness [17]. The planarians were nourished weekly with a fresh, organic beef liver homogenate. For experiments, planarians of 0.4–0.6 cm length were starved for at least 7 days prior to use. Regeneration was induced by excising both pre- and post-pharyngeal regions to produce head, trunk, and tail segments (Figure S1a), and only the trunk segments were used in this study. This dark condition was rigorously maintained throughout the entire study, including during regeneration, to standardize the environment and preclude any confounding effects of light.

### 2.2. Chemicals and IB Exposure

IB (CAS Number: 936563-96-1, purity ≥ 98%, Nanjing Baixindenuo Biotechnology Co., Ltd., Nanjing, China) was dissolved in dimethylsulfoxide (DMSO, CAS Number: 67-68-5, purity > 99.7%, Sigma-Aldrich, Taufkirchen, Germany) to prepare stock solution at a concentration of 81 g/L. This solution was stored at −20 °C and diluted to working concentrations with aerated tap water immediately prior to use. A vehicle control consisting of 0.1% DMSO was utilized, as this concentration has been previously demonstrated to exert no toxic effects on planarians [18].

According to the pre-experiment, a preliminary toxicity assessment was performed using a graded series of IB concentrations (1–6 mg/L, in 1 mg/L increments). The 96-h LC_50_ for *D. constrictiva* was determined to be 3.6 mg/L (Table S1). Following the initial observations of phenotypic responses, concentrations of 0.9 mg/L (1/4 of the LC_50_), 1.8 mg/L (1/2 of the LC_50_), and 2.7 mg/L (3/4 of the LC_50_) were selected for subsequent assays.

### 2.3. Morphological Observation and Locomotor Behavior Assessment

The morphological and regenerative effects of IB exposure were evaluated in both intact and regenerating planarians. Exposure to IB began 30 min post-amputation for regenerating planarians and immediately for intact ones (Figure S1b), following established protocols [19]. Morphological changes, survival rates, and regeneration progression were monitored daily using a Leica M165C stereomicroscope (Wetzlar, Germany). The regenerative capacity was quantitatively evaluated by analyzing the relative blastema size (defined as the ratio of blastema area to total body area) in thirty trunk fragments per experimental group, using ImageJ software (version 2.0.0, USA).

The effects of IB on planarian locomotor behavior were evaluated following 10 days exposure. Six planarians were randomly selected and transferred to a transparent Petri dish containing aerated tap water, placed atop a paper grid with 0.5 cm spacing. After a 30 s acclimation period, locomotion was recorded for 8 min. The locomotor behavior was analyzed using UMATracker software (Version: 2021/09/16) and ToxTrac tracking software (Version: 2.96 (2021/08/02)) to determine total distance traveled (mm), average speed (mm/s), and average acceleration (mm/s^2^) [16,20].

### 2.4. Whole-Mount Immunofluorescence

Whole-mount immunofluorescence was performed following the protocol of Jin et al. [21]. Planarians were processed for immunostaining using anti-SYNORF1 (3C11, 1:50, DSHB, AB_528479, The University of Iowa, Iowa City, IA, USA) to visualize the nervous system and anti-H3P (1:200, Millipore, Billerica, MA, USA, 06-570) to detect mitotic cells. Fluorescent images were acquired using a stereo fluorescence microscope (Axio Zoom V16, Zeiss, Jena, Germany). Analysis of brain dimensions was performed as previously described [22]. H3P-positive cells were counted using ImageJ software (version 2.0.0, Bethesda, MD, USA).

### 2.5. Whole-Mount in Situ Hybridization (WISH)

WISH was conducted following the established protocol by Jin et al. [23]. DIG-labeled RNA probes were synthesized via in vitro transcription using a commercial labeling kit (Roche, 11175025910, Basel, Switzerland), with T7 promoter sequences incorporated into the reverse primer during probe design (Table S2). Following the killing and removal of mucus in ice-cold 2% HCl [24], planarians were fixed in 4% paraformaldehyde (PFA). The subsequent processing involved bleaching with 6% H_2_O_2_ and treatment with proteinase K. Hybridization was performed at 56 °C for 16 h, followed by incubation with anti-DIG-AP (1:2000, Roche, 11093274910) and staining with NBT/BCIP (Roche, 11681451001).

### 2.6. Quantitative Reverse Transcription PCR (qRT-PCR)

Total RNA was isolated using RNAiso Plus (Takara, Dalian, China) following the established protocol [23]. The *ef2* gene was used as an internal control for mRNA expression normalization, with primer sequences designed using Primer 6.0 software (Table S2). Relative gene expression levels were determined from three biological replicates per sample using the 2^−ΔΔCt^ method.

### 2.7. Reactive Oxygen Species (ROS) Assay

ROS levels were detected using the fluorescent probe 2′,7′-dichlorofluorescein diacetate (DCFH-DA). Planarians were incubated in 10 μM DCFH-DA (1 mL) for 30 min prior to amputation and after exposure to IB under dark conditions. The amputated animals were then incubated in DCFH-DA for an additional 15 min. Following three washes with PBS buffer, the planarians were fixed in 1% low-melting-point agarose (Invitrogen) [25]. Photographs were captured using a fluorescence microscope (Axio Zoom V16, Zeiss, Germany), and quantification was conducted using ImageJ (version 2.0.0, Bethesda, MD, USA).

### 2.8. Western Blotting (WB)

Cleaved caspase-3 protein expression was analyzed using WB according to established protocols [19]. Immunoblotting was conducted with specific antibodies targeting Cleaved caspase-3 (C-Caspase-3, sc-7148, Santa Cruz Biotechnology, Inc., Santa Cruz, CA, USA) and β-Actin (sc-8432, Santa Cruz Biotechnology, Inc., Santa Cruz, CA, USA) as a loading control. Protein bands were detected by an ECL chemiluminescence kit (17,153, Millipore) and quantified using ImageJ software (version 2.0.0, Bethesda, MD, USA).

### 2.9. RNA Interference (RNAi) Experiments

Gene knockdown was achieved using bacterial-mediated RNA interference, as previously described [23]. The primers utilized were detailed in Table S2. Planarians were fed dsRNA-expressing bacteria ten times at two-day intervals, while control groups received non-targeting bacteria. Each RNAi experiment included at least of 30 planarians and was repeated for a minimum of three independent biological replicates. The knockdown efficiency was verified at both the transcriptional and protein levels by qRT-PCR, WISH, and WB.

### 2.10. Statistical Analysis

Statistical analyses were conducted using SPSS 26.0 and GraphPad Prism 9.0. The Shapiro–Wilk test was applied to assess data normality, and Levene’s test evaluated variance homogeneity, both using a significance threshold of α = 0.05. Significant differences among groups were determined using either one-way or two-way ANOVA followed by Tukey’s or Bonferroni’s post hoc tests for multiple comparisons, or an unpaired t-test with Welch’s correction. Effect sizes for ANOVA results are reported as partial eta-squared (η^2^). Concentration-response curves were fitted by nonlinear regression to determine EC_50_ values. Results are presented as means ± SD, with *p* < 0.05 considered statistically significant, and *p* < 0.01 considered highly significant.

## 3. Results

### 3.1. IB Exposure Induced Morphological Defects and Retarded Regeneration

**Figure 1:**
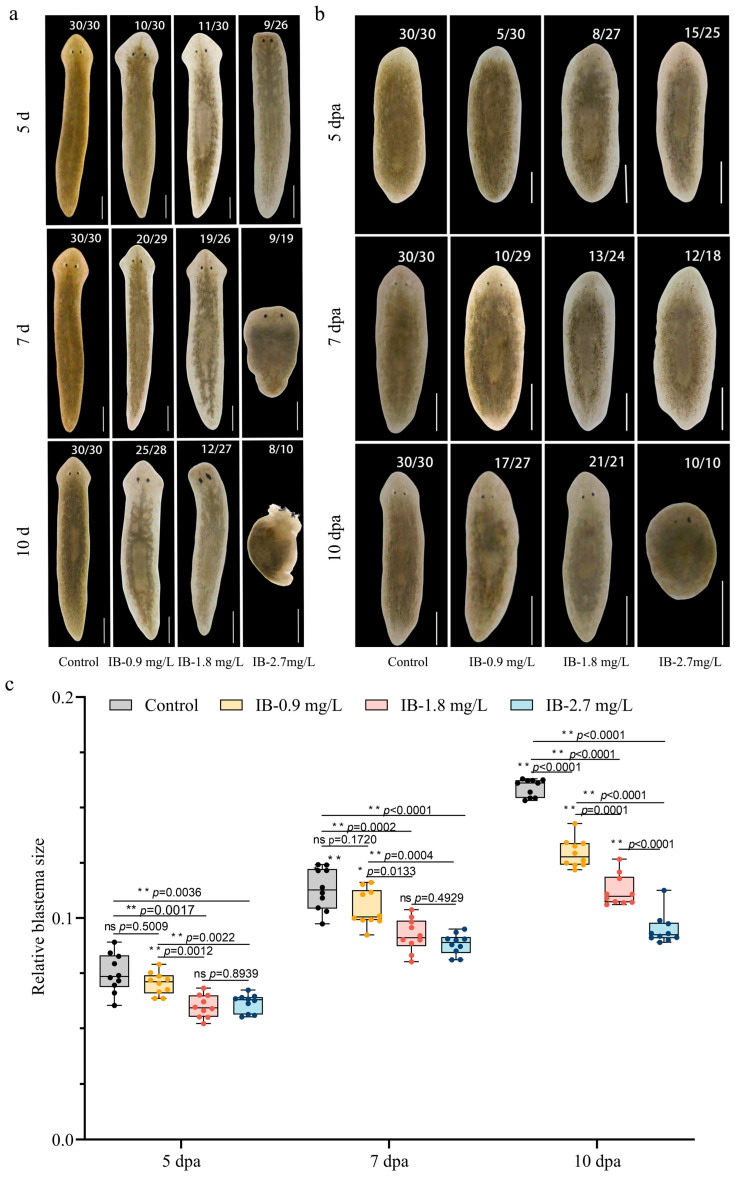
IB exposure induced morphological defects and retarded regeneration in planarians. (**a**,**b**) Representative images of morphological defects in intact (**a**) and regenerating (**b**) planarians. dpa: days post-amputation. n = 30, scale bar: 500 µm, the numbers with a slash (e.g., 9/26) denoted planarians with the phenotype per total survivors. (**c**) Quantification of relative blastema size in regenerating trunks. *p*-values were shown above the chart. Error bars represent mean ± SD (n = 10). ns, no significance, * *p* < 0.05, ** *p* < 0.01. *p*-values were calculated using a Two-way ANOVA with Tukey’s multiple comparison test. dpa: days post-amputation.

To evaluate the morphological changes induced by IB exposure, intact planarians and regenerating trunk fragments were subjected to concentrations of 0.9 mg/L (1/4 LC_50_), 1.8 mg/L (1/2 LC_50_), and 2.7 mg/L (3/4 LC_50_). Significant morphological changes were observed following to IB exposure. Initially, pigment anomalies, such as the appearance of pigment spots and a loss of body color, were evident in intact planarians exposed to all concentrations of IB. Prolonged exposure to 2.7 mg/L IB resulted in significant head regression, body swelling, and complete lysis (Figure 1a). IB exposure also impaired planarian regeneration in a time-dependent manner, causing a progressive delay in the process. Although the size of the blastema in planarians exposed to 0.9 mg/L IB did not differ significantly from that of control animals (*p* > 0.05), those exposed to 1.8 mg/L and 2.7 mg/L IB had significantly smaller blastemas compared to controls over a 10-day regeneration period (*p* < 0.01). Notably, the inhibitory effect on blastema size became progressively more pronounced over time. This pattern was substantiated by a two-way ANOVA, which revealed significant main effects of concentration (*F* (3, 36) = 147.9, *p* < 0.0001, partial η^2^ = 0.810) and time (*F* (2, 72) = 748.0, *p* < 0.0001, partial η^2^ = 0.931) on relative blastemas size, along with a significant concentration × time interaction (*F* (6, 72) = 27.61, *p* < 0.0001 partial, η^2^ = 0.601). The large effect sizes indicate that both factors and their interaction determined the outcome. Dose–response analysis further confirmed the time-dependent inhibitory effect of IB on blastema regeneration (Figure S2). Specifically, the EC50 values decreased systematically over time, from 10.19 mg/L (95% CI: 7.24–16.33) at 5 days post-anthesis (dpa) to 8.99 mg/L (95% CI: 6.96–12.39) at 7 dpa, and further to 4.10 mg/L (95% CI: 3.70–4.57) at 10 dpa. This progressive reduction in EC50 indicates a significant increase in the potency of IB during the later stages of regeneration. Moreover, the goodness-of-fit improved markedly over time, with the R^2^ value increasing from 0.47 at 5 dpa to 0.94 at 10 dpa, reflecting a more predictable concentration-response relationship as regeneration progressed. Concurrently, the proportion of lysed fragments increased with both increasing dose and prolonged exposure duration. Regenerated planarians also exhibited pigment anomalies, such as spotting and overall loss of body color (Figure 1b,c).

Collectively, these findings demonstrate that prolonging exposure to and increasing the concentration of IB enhanced its toxicity, which significantly disrupts planarian homeostasis and inhibited their regenerative capacities.

### 3.2. IB Exposure Impaired the Structure and Regeneration of CNS

The locomotor behavior of planarians was assessed after a 10-day exposure to IB. Control planarians, being photophobic, usually showed thigmotactic behavior, moving along container walls. In contrast, IB-exposed planarians had erratic movement patterns (Figure S3a). Quantitative analysis showed significant locomotor impairment in treated groups. Planarians exposed to 0.9 mg/L IB had shorter crawling paths than controls, and this effect was more obvious at 1.8 mg/L, with significant reductions in total distance traveled, mean speed, and mean acceleration (Figure S3b). These results indicate that IB exposure suppresses planarian locomotor activity.

**Figure 2:**
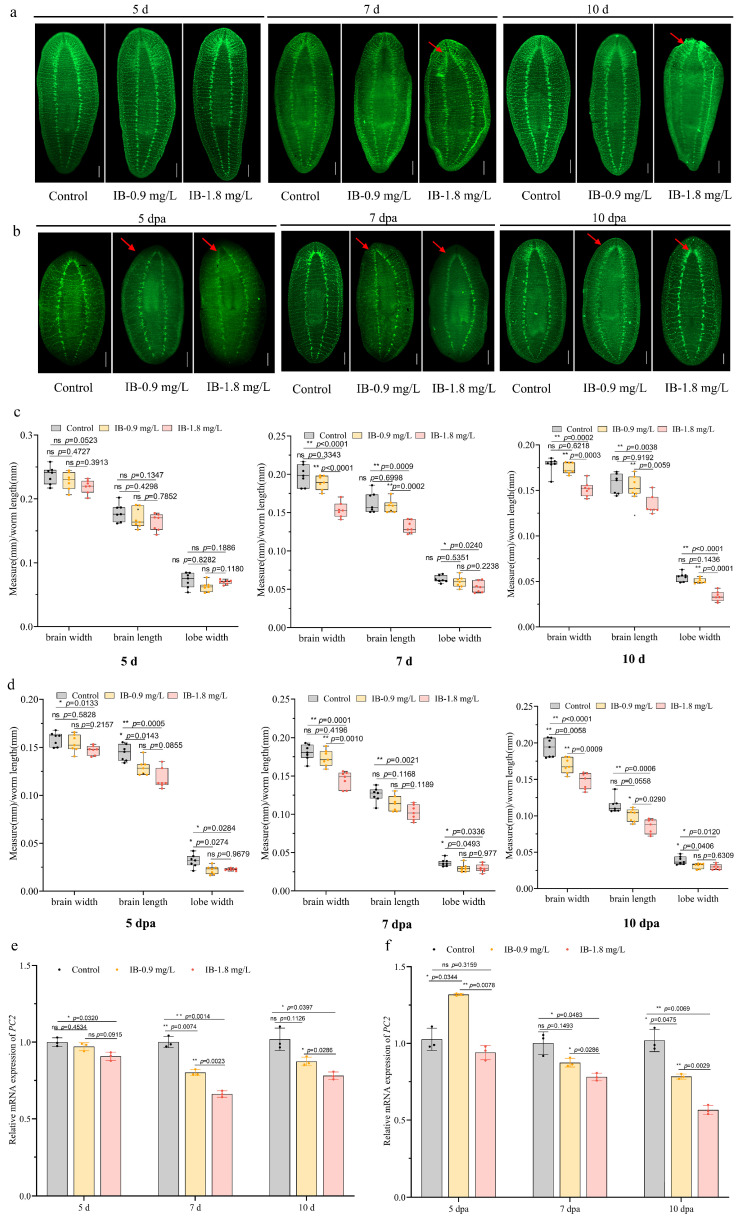
IB exposure impaired neural structure and inhibited neural regeneration in planarians. (**a**,**b**) Immunofluorescence staining of nervous system labeled with anti-SYNORF1 antibody in intact (**a**) and regenerating (**b**) planarians. Arrow: structural abnormalities. Scale bar: 200 µm. n = 7. (**c**,**d**) Quantification of normalized brain width, brain length, and lobe width in intact (**c**) and regenerating (**d**) planarians. *p*-values were shown above the chart. Error bars represent mean ± SD (n = 7). ns, no significance, * *p* < 0.05, ** *p* < 0.01. *p*-values were calculated using a Two-way ANOVA with Tukey’s multiple comparison test. (**e**,**f**) qRT-PCR analysis of pan-neuron marker gene *PC2* in intact (**e**) and regenerating (**f**) planarians. *p*-values were shown above the chart. Error bars represent mean ± SD (n = 3). ns, no significance, * *p* < 0.05, ** *p* < 0.01. *p*-values were calculated using a Two-way ANOVA with Tukey’s multiple comparison test. dpa: days post-amputation.

The pan-neuronal marker anti-SYNORF1 (3C11) targeting synapsin was used to label the whole planarian CNS [26]. Immunofluorescence staining with this antibody allowed us to analyze IB-induced structural changes in the nervous system. Due to high mortality rates at 2.7 mg/L IB and phenotypic similarities with 1.8 mg/L IB, subsequent analyses focused on 0.9 mg/L and 1.8 mg/L IB concentrations. Exposure to 0.9 mg/L IB did not significantly affect the planarian nervous system compared to controls on days 5, 7, or 10. In contrast, 1.8 mg/L IB induced time-dependent neurotoxicity. While no significant morphological differences were detected on day 5, the cephalic ganglia had regressed by day 7, manifested as significant reductions in brain length, width, and the brain lobe width to body length ratio. This degeneration progressed until the cephalic ganglia were nearly absent by day 10 (Figure 2a,b; Figure S4a,b).

In regenerative processes, control regenerates exhibited well-delineated, progressively enlarged cephalic ganglia and robust transverse commissures by day 5, forming a distinctive spongiform architecture by day 7. In contrast, exposure to either 0.9 or 1.8 mg/L IB resulted in dose-independent inhibition of nervous system regeneration. By day 5, animals exhibited markedly smaller, unconnected cephalic ganglia and underdeveloped ventral nerve cords with irregular commissures. This developmental impairment persisted, with only minimal ganglionic growth and open-ended ventral nerve cords visible by day 7. Ultimately, the cephalic ganglia remained underdeveloped even at day 10. Furthermore, quantitative morphometry confirmed that brain length, brain width, and the ratio of brain lobe width to body length were consistently and significantly diminished in planarians exposed to IB across the entire 10-day observation period (Figure 2c,d).

Quantitative analysis of the pan-neuronal gene *PC2* [16] confirmed significant down-regulation in both intact and regenerating planarians following a 10-day exposure to IB (Figure 2e,f). These results collectively indicate that IB exposure severely damages neural structure and markedly inhibits neural regeneration in planarians.

### 3.3. IB Exposure Disturbed Neoblast Proliferation and Differentiation

**Figure 3:**
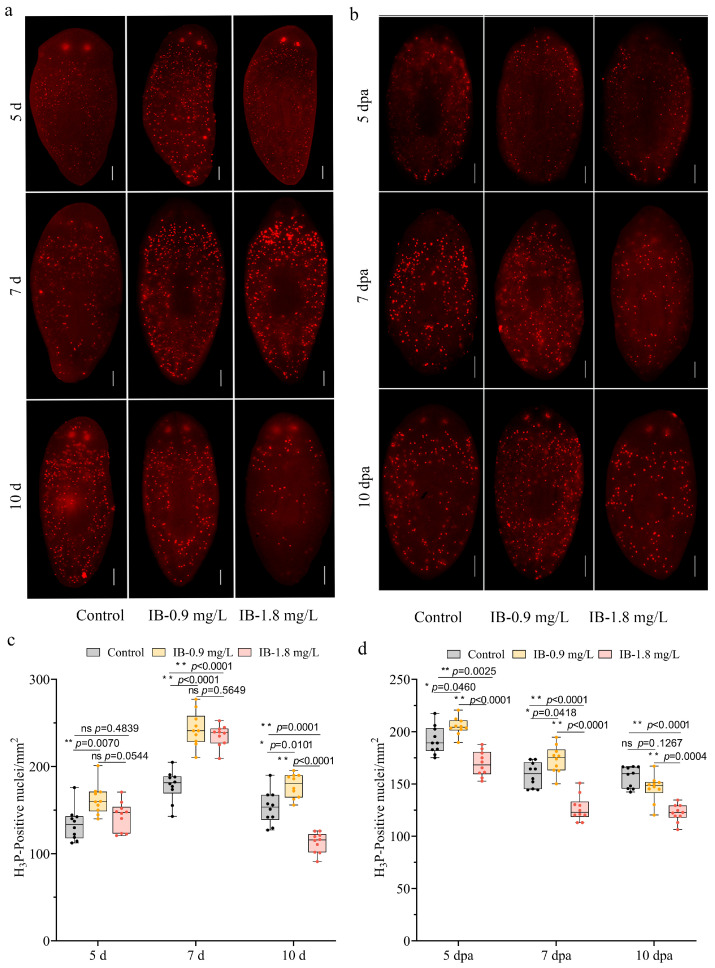
IB exposure disturbed neoblast proliferation in planarians. (**a**,**b**) Immunostaining of proliferating cells using anti-H3P antibody in intact (**a**) and regenerating (**b**) planarians. Scale bar: 200 µm. (**c**,**d**) Quantification of proliferating cells using anti-H3P antibody in intact (**c**) and regenerating (**d**) planarians. *p*-values were shown above the chart. Error bars represent mean ± SD (n = 10). ns, no significance, * *p* < 0.05, ** *p* < 0.01. *p*-values were calculated using a Two-way ANOVA with Tukey’s multiple comparison test. dpa: days post-amputation.

Neoblasts are the only mitotically active cell population in planarians and are essential for maintaining tissue homeostasis and driving regeneration through precisely regulated proliferation and differentiation [11]. To explore how IB disrupts planarian homeostasis and regeneration, the proliferative activity of neoblasts in both intact and regenerating planarians was investigated by immunofluorescence staining with an antibody against histone H3 phosphorylated at serine 10 (anti-H3P). In intact planarians, exposure to 0.9 mg/L IB resulted in a significant increase in H3P-positive cells on days 5, 7, and 10 compared to controls. In contrast, 1.8 mg/L IB elicited an early proliferative surge that peaked on day 7 but declined sharply by day 10 (Figure 3a,c). A two-way repeated measures ANOVA confirmed a significant interaction between time × concentration (*F* (4, 54) = 20.30, *p* < 0.0001, partial η^2^ = 0.530), along with significant main effects of time (*F* (2, 54) = 166.10, *p* < 0.0001, partial η^2^ = 0.826) and concentration (*F* (2, 27) = 61.38, *p* < 0.0001, partial η^2^ = 0.535), collectively demonstrating that IB disrupts neoblast proliferation in a concentration- and time-dependent manner in intact. In regenerating planarians, 0.9 mg/L IB enhanced proliferative activity on days 5 and 7 (*p* < 0.05), which returned to baseline levels by day 10 (*p* > 0.05). Conversely, 1.8 mg/L IB caused a sustained suppression of proliferation from day 5 onward, which persisted throughout the observation period (*p* < 0.01) (Figure 3b,d). The two-way repeated measures ANOVA again revealed a significant interaction between time and treatment (*F* (4, 54) = 5.02, *p* = 0.0026, partial η^2^ = 0.191), as well as main effects of time (*F* (2, 54) = 145.1, *p* < 0.0001, partial η^2^ =0.773) and concentration (*F* (2, 27) = 74.66, *p* < 0.0001, partial η^2^ = 0.671), further supporting the concentration- and time-dependent influence of IB on neoblast proliferation during regeneration.

**Figure 4:**
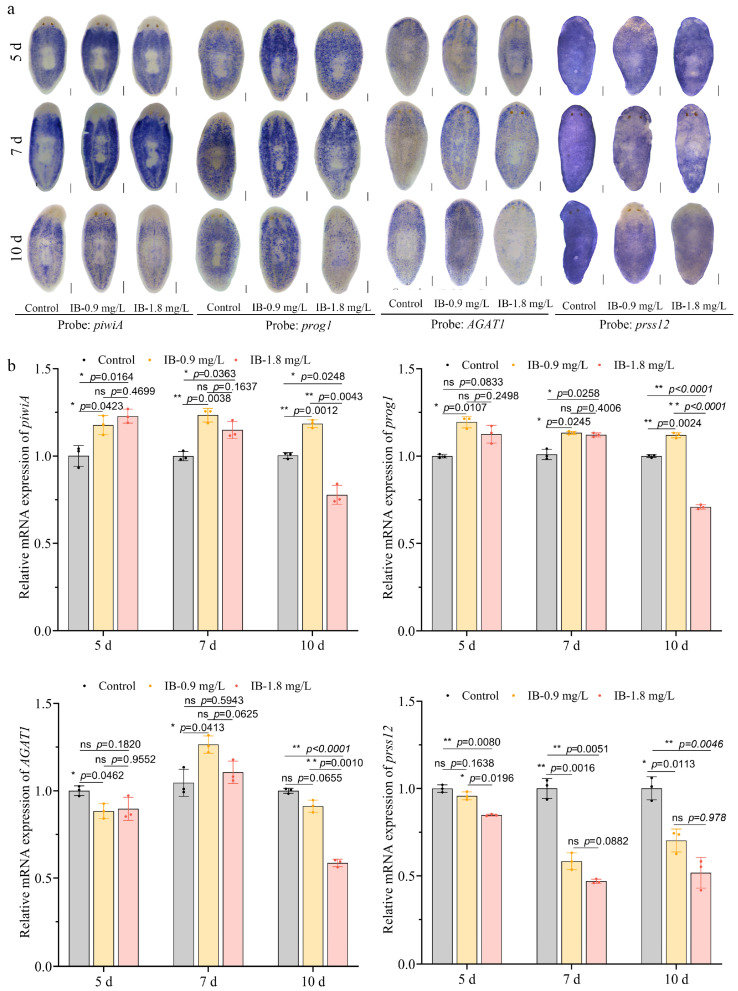
IB exposure disturbed neoblast differentiation into epidermal cells in intact planarians. (**a**) WISH analysis of epidermal lineage marker genes. Scale bar: 200 µm. n = 8. (**b**) qRT-PCR analysis of epidermal lineage marker genes. *p*-values were shown above the chart. Error bars represent mean ± SD (n = 3). ns, no significance, * *p* < 0.05, ** *p* < 0.01. *p*-values were calculated using a Two-way ANOVA with Tukey’s multiple comparison test.

**Figure 5:**
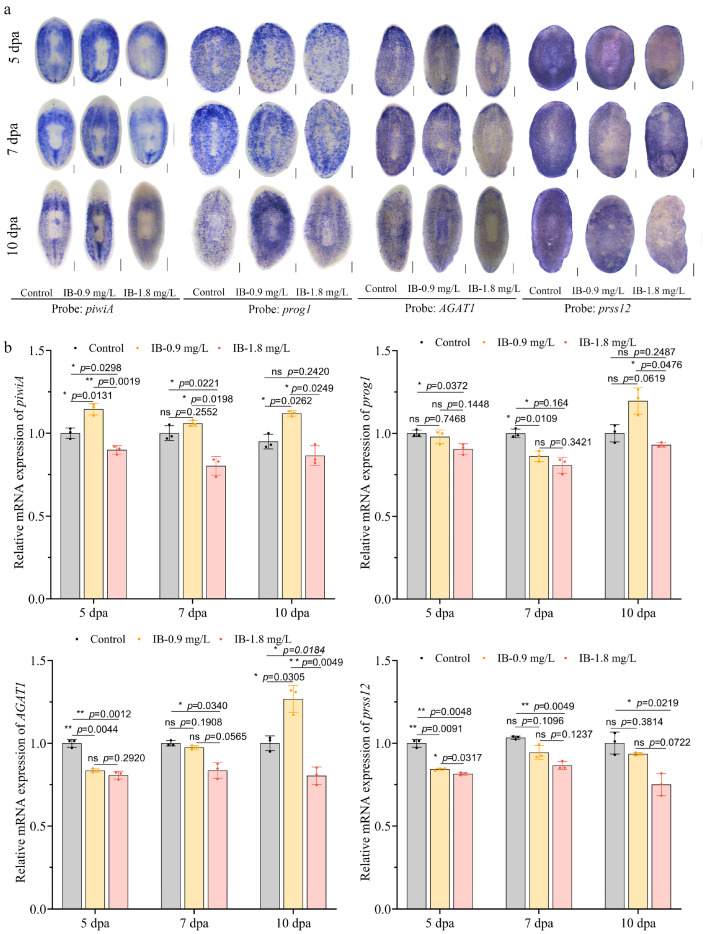
IB exposure disturbed neoblast differentiation into epidermal cells in regenerating planarians. (**a**) WISH analysis of epidermal lineage marker genes. Scale bar: 200 µm. n = 8. (**b**) qRT-PCR analysis of epidermal lineage marker genes. *p*-values were shown above the chart. Error bars represent mean ± SD (n = 3). ns, no significance, * *p* < 0.05, ** *p* < 0.01. *p*-values were calculated using a Two-way ANOVA with Tukey’s multiple comparison test. dpa: days post-amputation.

To evaluate the effects of IB on neoblast lineage differentiation, the epidermal differentiation lineage was initially investigated, which represents the most well-characterized neoblast differentiation pathway in planarians [27]. The WISH analysis revealed that 0.9 mg/L IB significantly upregulated the expression of the neoblast marker *piwiA*, the early epidermal progenitor marker *prog1*, and the late progenitor marker *AGAT1* in intact planarians. In contrast, 1.8 mg/L IB upregulated these markers initially, although levels subsequently declined over the exposure period. Notably, both concentrations significantly suppressed the expression of *prss12*, a mature epidermal cell marker (Figure 4a). These results were validated by qRT-PCR (Figure 4b). During regeneration, IB also impaired neoblast differentiation. Exposure to 0.9 mg/L IB transiently upregulated the epidermal differentiation markers *piwiA*, *prog1*, and *AGAT1* while suppressing *prss12*. However, this initial activation was overridden by prolonged exposure and higher doses, which ultimately led to the repression of all genes (Figure 5a,b).

To determine whether IB-induced pigment loss stems from defects in stem cell differentiation into pigment cells in planarians, the expression of the pigmentation regulator *Albino* and the pigment cell marker *PBGD* were analyzed [28,29]. In intact planarians, IB exposure dynamically modulated *Albino* expression, which remained unchanged at day 5, was significantly upregulated by day 7, and showed markedly downregulation at day 10. In contrast, *PBGD* expression exhibited a progressive, time- and dose-dependent decrease throughout the exposure period (Figure S5a,c). During regeneration, both genes showed a transient upregulation followed by significant downregulation with prolonged IB treatment. These effects were consistently dose-dependent (Figure S5b,d).

In summary, these findings suggest that IB exposure significantly disturbs neoblast proliferation and differentiation in planarians. These alterations may serve as the primary mechanistic basis for observed defects in homeostasis and regenerative capacity.

### 3.4. IB Exposure Led to Oxidative Stress and Excessive Apoptosis

**Figure 6:**
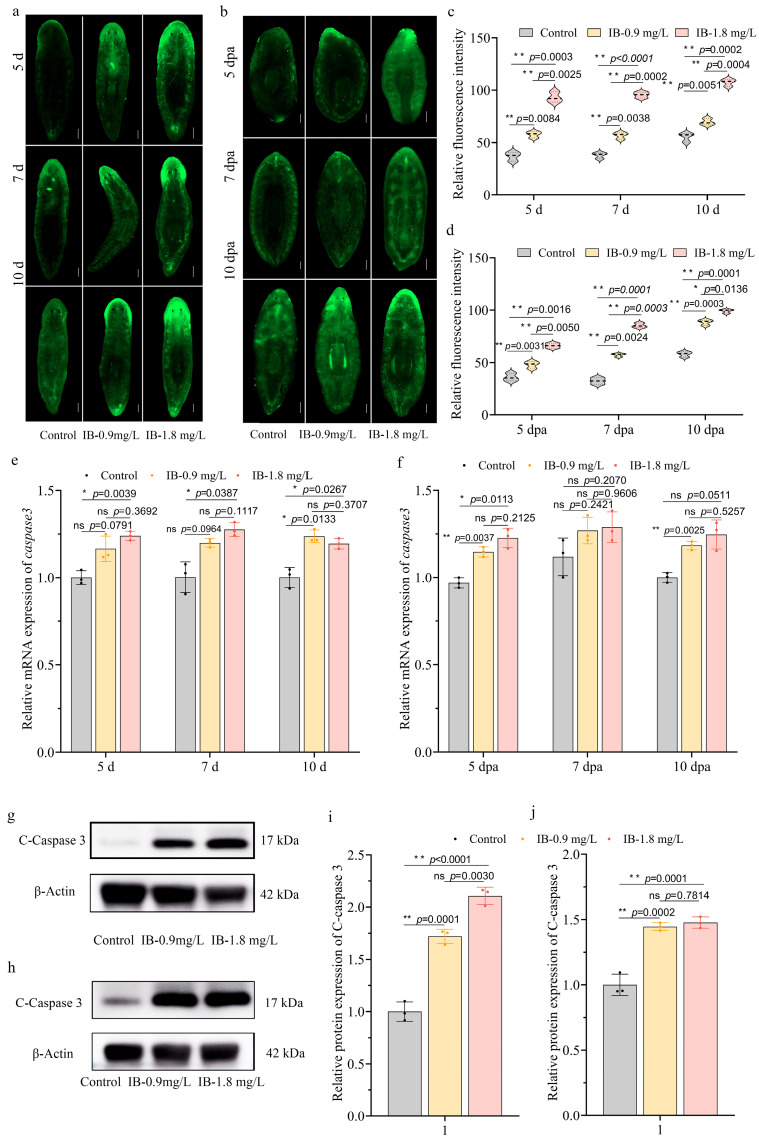
IB exposure led to oxidative stress and increased apoptosis in planarians. (**a**,**b**) Representative images of ROS levels in intact (**a**) and regenerating (**b**) planarians. Scale bar: 200 μm. n = 8. (**c**,**d**) Quantitative analysis of ROS levels in intact (**c**) and regenerating (**d**) planarians. *p*-values were shown above the chart. Error bars represent mean ± SD (n = 8). ns, no significance, * *p* < 0.05, ** *p* < 0.01. *p*-values were calculated using a Two-way ANOVA with Tukey’s multiple comparison test. (**e**,**f**) qRT-PCR analysis of *caspase3* in intact (**e**) and regenerating (**f**) planarians. *p*-values were shown above the chart. Error bars represent mean ± SD (n = 3). ns, no significance, * *p* < 0.05, ** *p* < 0.01. *p*-values were calculated using a Two-way ANOVA with Tukey’s multiple comparison test. (**g**,**h**) WB detection of Cleaved Caspase-3 protein expression in intact (**g**) and regenerating (**h**) planarians. (**i**,**j**) Quantification of WB results in intact (**i**) and regenerating (**j**) planarians. *p*-values were shown above the chart. Error bars represent mean ± SD (n = 3). ns, no significance, * *p* < 0.05, ** *p* < 0.01. *p*-values were calculated using a One-way ANOVA using Tukey’s multiple comparison test. dpa: days post-amputation. Original images can be found in Supplementary Materials.

Oxidative stress, resulting from an imbalance between the reactive oxygen species (ROS) production and cellular antioxidant defenses, serves as a pivotal mechanism underlying drug-induced biotoxicity by compromising redox homeostasis [16]. In this study, ROS levels were quantified in vivo by measuring the green fluorescence of the DCFH-DA probe, whose intensity is directly proportional to ROS abundance. The results indicated that exposure to 0.9 mg/L and 1.8 mg/L of IB resulted in a significant, dose- and time-dependent increase in ROS levels in both intact and regenerating planarians (Figure 6a,b). Statistical analysis confirmed this markedly increased compared to controls on days 5, 7, and 10 (*p* < 0.01) (Figure 6c,d), indicating a significant impairment of antioxidant defenses.

Excessive ROS accumulation can trigger apoptotic pathways, which is a well-established mechanism [30]. In our study, exposure to 0.9 mg/L and 1.8 mg/L IB resulted in a significant dose-dependent upregulation of *caspase3*, a central executioner of apoptosis [31], on days 5, 7, and 10 in both intact and regenerating planarians (Figure 6e,f). Furthermore, WB analysis confirmed an increase in activated caspase-3 (cleaved Caspase-3, C-caspase 3) protein levels at day 10 in planarians exposed to IB compared to controls (Figure 6g–j). These findings collectively demonstrate that IB exposure elevates ROS levels, resulting in oxidative damage and subsequent activation of apoptotic pathways, which likely disturbs tissue homeostasis and cellular renewal processes, ultimately compromising both homeostasis and regenerative capacity in planarians.

### 3.5. IB Exposure Downregulated BTK Expression

**Figure 7:**
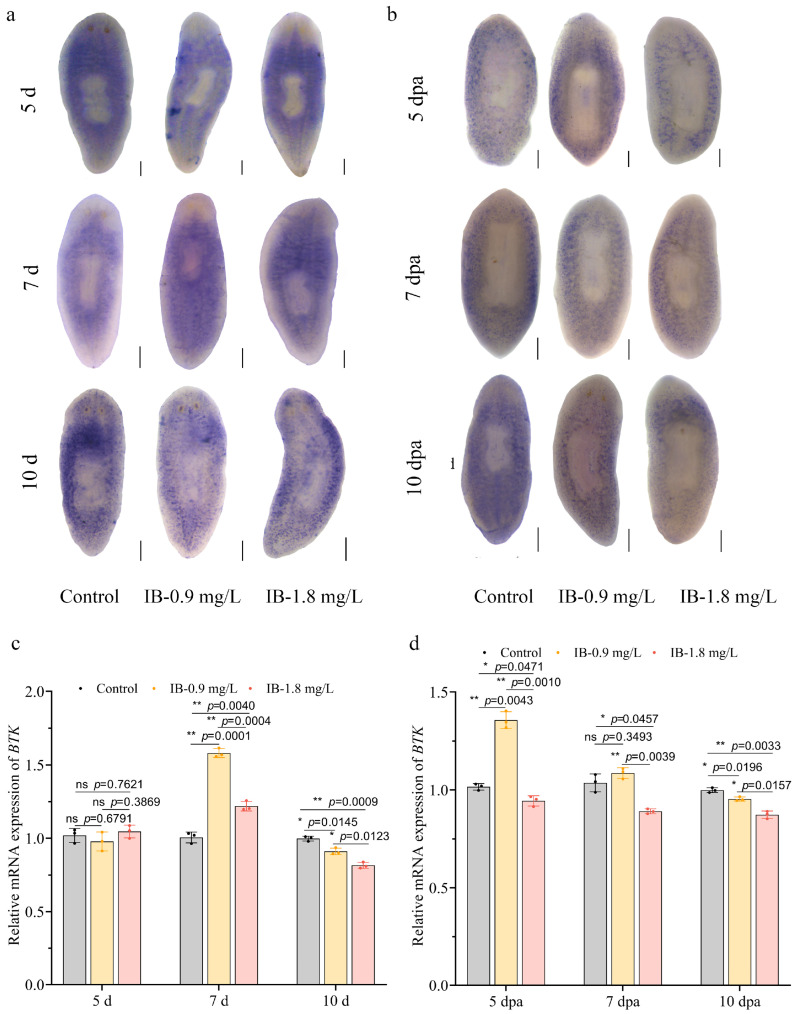
IB exposure downregulated *BTK* expression in planarians. (**a**,**b**) WISH analysis of *BTK* mRNA levels in intact (**a**) and regenerating (**b**) planarians. Scale bar: 200 µm. n = 8. (**c**,**d**) WISH analysis of *BTK* mRNA levels in intact (**c**) and regenerating (**d**) planarians. *p*-values were shown above the chart. Error bars represent mean ± SD (n = 3). ns, no significance, * *p* < 0.05, ** *p* < 0.01. *p*-values were calculated using a Two-way ANOVA with Tukey’s multiple comparison test. dpa: days post-amputation.

To elucidate the molecular basis of toxicity induced by IB in planarians, a *BTK* homolog was first identified and sequenced from *D. constrictiva*. Domain architecture analysis showed that the predicted protein has all characteristic domains of the BTK family (N-terminal PH domain, followed by SH3, SH2, and C-terminal protein kinase domains), indicating high conservation with BTKs from other species like *Schmidtea mediterranea* and *Dugesia japonica*. This confirmed its identity and allowed subsequent expression analysis (Figure S6). The temporal dynamics of *BTK* expression were then characterized in both intact and regenerating planarians following IB exposure. WISH analysis revealed no significant alteration in *BTK* expression after 5 days of exposure (*p* > 0.05). However, a significant upregulation was observed by day 7 (*p* < 0.01), followed by significant down-regulation after prolonged exposure to day 10 in intact planarians (*p* < 0.01) (Figure 7a). This regulatory dynamic was consistently confirmed by qRT-PCR measurements (Figure 7c).

During regeneration, *BTK* expression exhibited a more complex regulatory pattern in response to IB exposure. At 0.9 mg/L IB, *BTK* expression levels showed a transient elevation at day 5 (*p* < 0.01), returned to baseline by day 7, and displayed a not statistically significant downward trend by day 10 (*p* > 0.05). In contrast, 1.8 mg/L IB induced a marked suppression of *BTK* expression at day 5 (*p* < 0.05), with sustained inhibition throughout days 7 and 10 (*p* < 0.01) (Figure 7b,d). Taken together, these results suggest that *BTK* signaling inhibition may contribute to the toxicological effects of IB in planarians.

### 3.6. BTK RNAi Caused Morphological and Regenerative Defects

**Figure 8:**
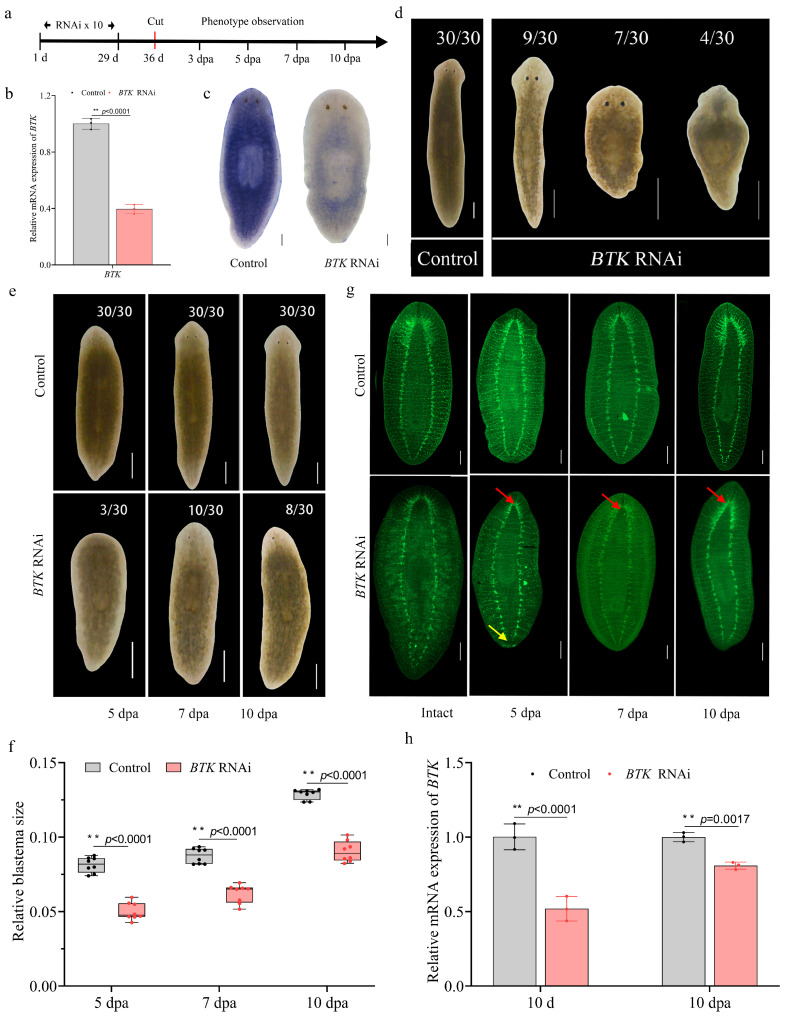
*BTK* RNAi caused morphological, regenerative and neural defects in planarians. (**a**) Schematic diagram of the *BTK* RNAi procedure. Cut: the time point when the planarian was amputated to generate regenerating fragments. (**b**) qRT-PCR analysis of *BTK* mRNA expression levels after *BTK* RNAi. *p*-values were shown above the chart. Error bars represent mean ± SD (n = 3). ** *p* < 0.01. *p*-values were calculated by an unpaired t test with Welch’s correction. (**c**) WISH analysis of *BTK* mRNA expression levels after *BTK* RNAi. Scale bar: 200 µm. n = 8. (**d**,**e**) Representative images of morphological defects in intact (**d**) and regenerating (**e**) planarians after *BTK* RNAi. Scale bar: 500 µm, n = 30, the numbers with a slash (e.g., 9/30) denoted planarians with the phenotype per total survivors. (**f**) Quantification of relative blastema size in regenerating trunk fragments after *BTK* RNAi. *p*-values were shown above the chart. Error bars represent mean ± SD (n = 8). ** *p* < 0.01. *p*-values were calculated using a Two-way ANOVA with Bonferroni’s multiple comparisons test. (**g**) Nervous system labeled with anti-SYNORF1 antibody in intact and regenerating planarians after *BTK* RNAi. Red arrow: abnormal structure in the regenerated head. Yellow arrow: abnormal structure in the regenerated tail. Scale bar: 200 µm. n = 8. (**h**) qRT-PCR analysis of *PC2* (pan-neuronal marker) expression in intact and regenerating planarians after BTK RNAi. *p*-values were shown above the chart. Error bars represent mean ± SD (n = 3). ** *p* < 0.01. *p*-values were calculated using a Two-way ANOVA with Bonferroni’s multiple comparisons test. dpa: days post-amputation.

Based on the significant suppression of *BTK* expression following IB exposure and the existing evidence that IB exerts pharmacological effects through *BTK* inhibition [3], we hypothesize that this conserved mechanism may underlie its toxicological effects in planarians. To test this hypothesis, a *BTK* knockdown model was established by feeding planarians *Escherichia coli* HT115 expressing *BTK* dsRNA 10 times (Figure 8a). qRT-PCR and WISH analyses confirmed that *BTK* expression was significantly downregulated in *BTK* RNAi planarians compared to controls on day 7 after the final RNAi feeding (*p* < 0.01) (Figure 8b,c).

*BTK* RNAi caused distinct morphological and regenerative defects in planarians. Intact planarians exhibited pigmentation loss (9/30), body swelling (7/30), and tissue lysis (4/30) (Figure 8d). Regeneration assays revealed severe regenerative defects in planarians following *BTK* RNAi (Figure 8e). On day 3 of regeneration, the blastemas were notably smaller with whitened body margins (2/30). By day 5, regeneration became more suppressed with complete body whitening (2/30), followed by the appearance of asymmetrical eyespots on day 7 (6/30). The phenotypic defects continued to worsen through day 10. Quantification of blastema size supported that *BTK* RNAi inhibited planarian regeneration (Figure 8f).

Immunostaining with the anti-SYNORF1 antibody revealed significant neuroanatomical disruptions in planarians following *BTK* RNAi (Figure 8g). Compared to the controls, the cephalic ganglia in intact planarians following *BTK* RNAi were indistinct, the ventral nerve cords were discontinuous, and the transverse commissures appeared vague and irregular. During regeneration, notable differences in nervous system regeneration were observed between control and *BTK* RNAi planarians. By day 5, the cephalic ganglia remained indistinct and the ventral nerve cords had failed to close completely in *BTK* RNAi animals. In contrast, control planarians displayed clearly defined cephalic ganglia and well-organized transverse commissures by day 7. *BTK* RNAi planarians, however, exhibited significantly smaller and underdeveloped cephalic ganglia at this stage. By day 10, the cephalic ganglia in *BTK* RNAi specimens remained poorly structured compared to the well-differentiated ganglia in control animals. qRT-PCR results revealed a significant downregulation of *PC2* expression in both intact and regenerating planarians on day 10 following *BTK* RNAi (Figure 8h). These results demonstrate that *BTK* RNAi leads to morphological and regenerative defects, including disrupted neural integrity and impaired regeneration, which are similar to the effects of IB exposure, suggesting that *BTK* mediates IB-induced toxicity in planarians as a key molecular target.

### 3.7. BTK RNAi Disturbed Neoblast Proliferation and Differentiation, and Increased Apoptosis

**Figure 9:**
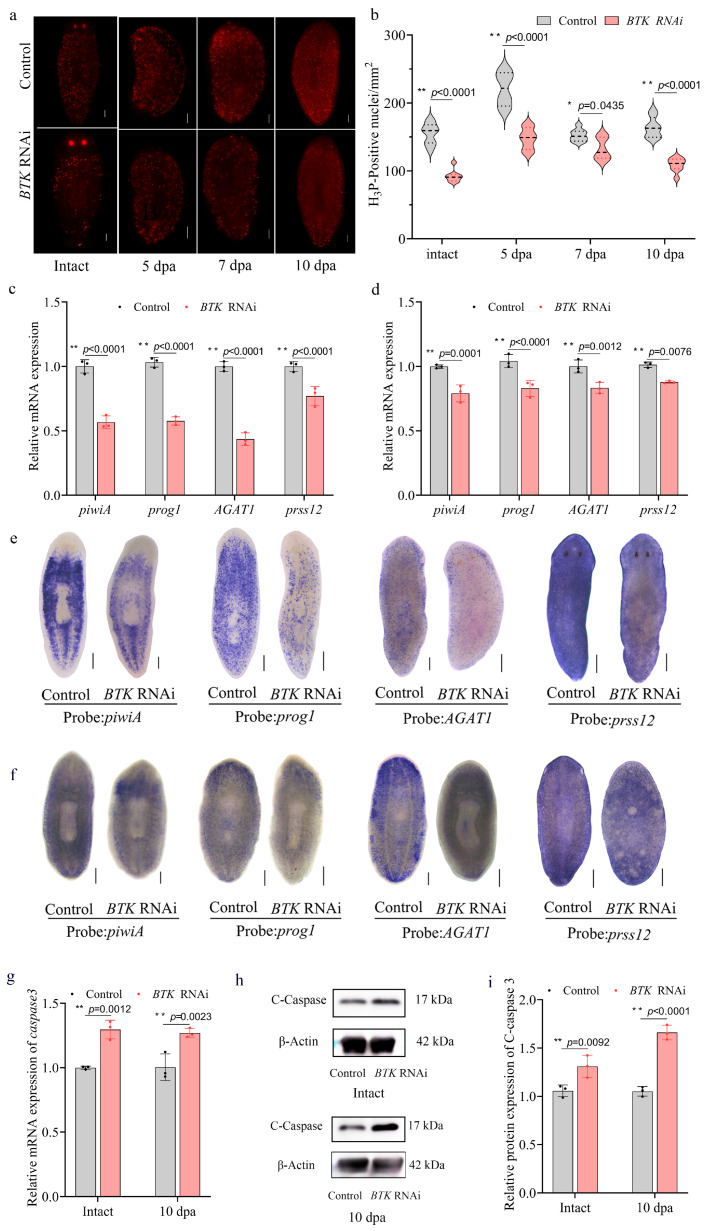
*BTK* RNAi suppressed neoblast proliferation and differentiation and induced apoptosis in planarians. (**a**,**b**) Anti-H3P immunostaining (**a**) and its quantification (**b**) showing cell proliferation in intact and regenerating planarians. (**c**,**d**) qRT-PCR analysis of epidermal differentiation gene expression in intact (**c**) and regenerating (**d**) planarians. *p*-values were shown above the chart. Error bars represent mean ± SD (n = 3). * *p* < 0.05, ** *p* < 0.01. *p*-values were calculated using a Two-way ANOVA with Bonferroni’s multiple comparisons test. (**e**,**f**) WISH analysis of epidermal differentiation gene expression in intact (**e**) and regenerating (**f**) planarians. *p*-values were shown above the chart. Scale bar: 200 µm. n = 8. (**g**) qRT-PCR analysis of *caspase3* expression in intact and regenerating planarians. *p*-values were shown above the chart. Error bars represent mean ± SD (n = 3). * *p* < 0.05, ** *p* < 0.01. *p*-values were calculated using a Two-way ANOVA with Bonferroni’s multiple comparisons test. (**h**,**i**) WB (**h**) and quantitative analysis (**i**) of Cleaved Caspase-3 protein levels in intact and regenerating planarians. *p*-values were shown above the chart. Error bars represent mean ± SD (n = 3). ** *p* < 0.01. *p*-values were calculated using a Two-way ANOVA with Bonferroni’s multiple comparisons test. dpa: days post-amputation. Original images can be found in Supplementary Materials.

To assess the effect of *BTK* RNAi on neoblast proliferation, proliferating cells were detected by immunofluorescence staining using anti-H3P antibody in both intact and regenerating planarians. The results showed a significant reduction in the number of H3P-positive cells following *BTK* RNAi compared to the control group (*p* < 0.01). During regeneration, *BTK* RNAi planarians showed persistently lower numbers of H3P-positive cells at days 5, 7, and 10 post-amputation (*p* < 0.01) (Figure 9a,b).

The analysis of the differentiation of the epidermal lineage revealed a significant downregulation of *piwiA*, *prog1*, *AGAT1*, and *prss12* mRNA in intact planarians following *BTK* RNAi (*p* < 0.01) (Figure 9c). WISH analysis verified these results, showing a marked downregulation in the spatial expression of all genes examined (Figure 9e). The transcriptional suppression and spatially restricted expression of epidermal lineage differentiation genes were also observed in 10-day regenerating specimens (Figure 9d,f). In addition, *BTK* RNAi significantly impaired pigmentation by disrupting stem cell differentiation into pigment cells. Expression of the key pigmentation regulator *Albino* was markedly reduced in both intact and regenerating planarians. Notably, the characteristic signal enrichment in head and tail blastemas drastically reduced during regeneration. Similarly, the punctate pattern of pigment cell marker *PBGD* became significantly sparser following *BTK* RNAi, and regenerating blastemas displayed a substantial reduction in *PBGD* puncta (Figure S7).

Furthermore, *BTK* RNAi significantly upregulated *caspase-3* expression in intact and regenerating planarians (*p* < 0.01) (Figure 9g). Corresponding WB analyses revealed an increase in C-caspase-3 levels in these planarians compared to control animals (*p* < 0.01) (Figure 9h,i). These results indicate that *BTK* RNAi suppresses neoblast proliferation and differentiation, while also promoting cell apoptosis in planarians.

Collectively, these findings demonstrate that *BTK* RNAi parallels the regenerative defects and stem cell dynamics disruptions induced by IB exposure. This provides direct support for the hypothesis that IB may exert its toxic effects primarily by inhibiting *BTK* in planarians.

## 4. Discussion

This study presents the first systematic assessment of the developmental toxicity of IB and its underlying mechanisms using the freshwater planarian *D. constrictiva*, a powerful invertebrate model for stem cell biology and regeneration research. Although the genomic resources for *D. constrictiva* are not as comprehensive as those for other established planarian models, our functional data strongly support its usefulness in toxicological evaluation. Our findings show that IB induces potent effects at both organismal and cellular levels, leading to severe morphological defects, regeneration delays, and neural regeneration impairment. Importantly, we observed significant structural abnormalities and neurobehavioral deficits in planarians exposed to IB, including dose-dependent reductions in locomotor velocity and overall motility, which functionally validated the neurotoxicity induced by IB. Mechanistic studies further demonstrated that IB disrupts the neoblast dynamic by inhibiting proliferation and differentiation, and triggering oxidative stress-mediated apoptosis. The observed oxidative imbalance indicates redox homeostasis disruption, potentially involving the downregulation of key antioxidant enzymes such as superoxide dismutase (SOD), catalase (CAT), and glutathione peroxidase (GPx) [16,32], a crucial direction for future validation. While direct biochemical confirmation of BTK inhibition (e.g., phosphorylation status or downstream signaling) remains challenging in this model due to a lack of species-specific reagents, our functional genetic evidence strongly supports that *BTK* inhibition may serve as a central mechanism underlying these toxic effects.

Our observations extend and reinforce previous reports of the developmental toxicity of IB. The dose-dependent induction of morphological defects, including head regression, body lysis, neural deformities, and blastema hypoplasia, closely parallels the teratogenic effects reported in mammalian and zebrafish models, such as fetal growth restriction, visceral malformations, and neural tube defects [9]. This evolutionary conservation of toxic phenotypes underscores the fundamental role of BTK across species. Notably, significant toxicity was observed in planarians at concentrations as low as 0.9 mg/L, which is substantially below the steady-state plasma level in patients receiving 420 mg/day IB (~29.5 mg/L) [33] and also lower than the toxic threshold in zebrafish (~5 μM or 2.2 mg/L) [10]. To better understand the toxic potency of IB, it was compared with CdCl_2_, a classic reference toxicant. The 96-h LC_50_ of IB in *D. constrictiva* was 3.6 mg/L. While literature lacked an exact LC_50_ for CdCl_2_, exposure of *Dugesia dorotocephala* to 3.2 mg/L CdCl_2_ resulted in 100% mortality in two weeks, indicating that the acute lethality of IB is comparable to that of the well-known potent heavy metal toxicant, CdCl_2_ [34]. This remarkable sensitivity highlights the value of planarians as predictive bioindicators and underscores the need to reassess potential low-dose toxicity risks of IB, particularly in developing tissues.

The maintenance of tissue homeostasis and regeneration critically depends on the tightly coordinated regulation of neoblast proliferation, differentiation, and apoptosis [11]. Our results demonstrate that IB disrupts these essential stem cell processes, thus leading to developmental toxicity. An initial transient proliferative surge, marked by upregulation of *piwiA* and *prog1*, potentially representing a compensatory response to epidermal damage or mild stress, similar to stress-induced proliferation observed in other models, such as in the zebrafish vascular system [10]. However, prolonged or high-dose IB exposure exceeded this adaptive capacity, resulting in sustained suppression of neoblast proliferation and a global downregulation of differentiation markers across epidermal, pigment, and other lineages. This ultimately led to a loss of functional stem cells. Moreover, this loss was made worse by a strong increase in cell apoptosis, which was caused by too much ROS. The resulting oxidative stress disrupted the critical balance among proliferation, differentiation, and cell death, a triad indispensable for regeneration and tissue homeostasis, thereby causing severe regeneration delays and loss of structural integrity. While the precise causal sequence between BTK inhibition and the observed oxidative stress warrants further investigation, the functional concordance between chemical inhibition by IB and genetic knockdown of *BTK* in recapitulating the entire phenotypic spectrum, including ROS accumulation and apoptosis, strongly argues that oxidative stress is a central component of the toxicity mechanism rather than an epiphenomenon. Future studies employing antioxidant rescue assays or targeting downstream signaling nodes in more pharmacologically tractable models will be valuable to further dissect this causal relationship. These findings align with reports that BTK inhibitors induce oxidative stress and caspase-dependent apoptosis in mammalian cells [35,36], underscoring the potential of IB to disrupt evolutionarily conserved developmental processes.

A key contribution of this study is the direct linkage of IB-induced toxicity to *BTK* inhibition within the context of stem cell biology and regeneration. Our findings demonstrate that IB exposure downregulated *BTK* expression, and *BTK* RNAi paralleled the spectrum of IB-induced defects, including the morphological, regenerative, proliferative, and apoptotic effects. This functional convergence identifies *BTK* as the primary molecular target responsible for IB’s developmental toxicity. As a member of the TEC family of tyrosine kinases, BTK modulates cell survival and differentiation through core signaling pathways including PI3K-AKT, NF-κB, and MAPK cascades [37]. We propose that IB-induced BTK inhibition disrupts homeostasis mainly through the BTK-PI3K-AKT-NF-κB signaling axis, impairing cell survival and differentiation. The observed oxidative stress may result from this signaling disruption and amplify the apoptotic signal, creating a vicious cycle that leads to the failure of stem cell maintenance and tissue regeneration. This mechanism conserved across metazoans. Accordingly, parallel phenotypes have been documented in BTK-deficient mice [38], during CAR-T cell production [39], and in HER2-positive breast cancer stem cells [40], highlighting the broad role of BTK in cell fate determination. Importantly, the toxicity of BTK inhibition appears to be highly compound-specific. Preliminary observations suggest developmental toxicity severity may correlate with inhibitor selectivity. Next-generation inhibitors like acalabrutinib, with higher specificity, have a reduced toxic impact compared to IB in the planarian model. This evolutionary conservation implies that the therapeutic efficacy of BTK inhibition may inevitably involve a compromise in regenerative capacity. Thus, the therapeutic mechanism of IB is fundamentally linked to its damaging effects on stem cells and developing tissues.

Beyond that, this study also demonstrates that IB induces observable neurotoxicity and impairs neuroregeneration in planarians. These effects were evidenced by dose-dependent loss of cephalic ganglia, impaired regeneration of ventral nerve cords and transverse commissures, reduced brain size, and downregulation *PC2* expression, collectively indicating severe damage to existing neural architecture and compromised neural regeneration. The observed locomotor deficiencies corroborate these structural neural impairments, demonstrating a direct functional consequence of the neurodevelopmental damage. Given the evolutionary conservation of BTK signaling and its established role in neurogenesis [2], IB likely mediates these effects by interfering with fundamental processes such as neural stem cell differentiation, axon guidance, or synapse formation. While results offer insights into potential developmental neurotoxicity of BTK inhibition, direct extrapolation to human clinical contexts needs caution due to physiological differences between planarians and mammals. The physiological and pharmacokinetic environment in planarians differs from that of mammals, which can change a drug’s bioavailability, metabolism, and toxicity. So, the concentrations used here show the drug’s ability to disrupt processes like stem cell dynamics and neurogenesis, not predict human toxic doses. However, the observed neural vulnerability in the model is clinically relevant when combined with reports of neurological adverse effects in some patients treated with IB [37]. The convergence of experimental findings and clinical observations suggests BTK signaling may play an under-appreciated role in neural development, needing further investigation. This is especially relevant for pediatric populations, as the recent approval of IB for cGVHD and its off-label use highlight the need for careful neurodevelopmental monitoring [7,8]. Interestingly, clinical studies present a complex and seemingly contradictory profile: IB shows neuroprotective effects in conditions such as CNS lymphoma [41] and neuroinflammatory models [42], yet also correlates with neuropathy in some patients [37]. This apparent contradiction highlights the context-dependent nature of the neurobiological effects of IB, which may potentially be therapeutic in cases of immune dysregulation, but harmful to normal development and regeneration. Consequently, further mechanistic and translational studies are essential to elucidate IB’s complex role in neural integrity and repair.

However, there are several limitations to our study. First, planarians, although a powerful model for regeneration biology, differ substantially from vertebrates in physiology, pharmacokinetics, and immune system complexity, which restricts direct clinical extrapolation. Second, we focused on acute exposure scenarios. Future studies using chronic, low-dose exposures are needed to better model the potential effects of prolonged clinical administration or environmental exposure and evaluate subtler, sublethal toxicological endpoints. Third, we have integrated locomotor behavior assessment, and additional functional evaluations like light response and feeding behavior may provide more insights into specific neural circuit impairments. The lack of direct biochemical measurements, e.g., BTK phosphorylation or downstream signaling activity, highlights methodological limitations when working with non-traditional model organisms lacking species-specific reagents. Fourth, our mechanistic insights are derived from a targeted, hypothesis-driven approach. While this provides focused evidence, it does not encompass all molecular pathways affected by IB. Future global transcriptomic or proteomic analyses, a separate and comprehensive research effort, are needed to uncover broader network perturbations. Finally, while our study provides a basic understanding of IB toxicity in planarians, further characterization of next-generation BTK inhibitors in this model can clarify how kinase selectivity affects developmental toxicity. Addressing these limitations in future work, especially by extending studies to vertebrate developmental models and human organoid systems, is essential to validate the translational relevance of our findings.

## 5. Conclusions

In conclusion, our study demonstrates that IB induces morphological and regenerative defects in planarians through *BTK* inhibition, which disrupts stem cell dynamics by impairing proliferation, blocking differentiation, and triggering apoptosis via ROS-mediated pathways. Critically, the concordance between pharmacological and genetic inhibition provides robust functional validation of *BTK*’s central role in these processes. Although direct clinical extrapolation requires validation in mammalian models, our findings suggest that neurocognitive assessments, oxidative stress biomarkers, and tissue-specific regenerative indicators warrant investigation in patients undergoing BTK inhibitor therapy, particularly in pediatric populations. These insights provide a foundation for optimizing therapeutic strategies that balance therapeutic efficacy against developmental safety considerations, particularly for vulnerable populations requiring long-term treatment.

## Data Availability

The original contributions presented in this study are included in the article/supplementary material. Further inquiries can be directed to the corresponding author.

## Supplementary Materials

The following supporting information can be downloaded at: https://www.mdpi.com/article/10.3390/biom15121665/s1, Figure S1: Schedule of the experimental procedure. (a) Schematic illustration of planarian amputation. Dashed line: transverse amputation site. Scale bar: 500 µm. (b) Schematic overview of the drug exposure protocol in regenerating planarians. dpa: days post-amputation. Figure S2: Dose-response curve of IB inhibition on blastema size. Figure S3: IB exposure changed locomotor behavior in planarians. (a) Movement trajectories. (b) Analysis of total movement distance, average speed and average acceleration. The units of distance, speed and acceleration was mm, mm s-1, and mm s-2, respectively. *p*-values were shown above the chart. Error bars represent mean ± SD (n = 3). ** *p* < 0.01. *p*-values were calculated using a One-way ANOVA with Tukey’s multiple comparison test. Figure S4: A higher magnification view of neural structural abnormalities in intact (a) and regenerating (b) planarians exposed to IB. Arrow: structural abnormalities. Scale bar: 200 µm. dpa: days post-amputation. Figure S5: IB exposure disturbed the stem cell differentiation into pigment cells in planarians. (a,b) WISH analysis of stem cell differentiation into pigment cells in intact (a) and regenerating (b) planarians. Scale bar: 200 µm. n = 8. (c,d) qRT-PCR analysis of stem cell differentiation into pigment cells in intact (c) and regenerating (d) planarians. *p*-values were shown above the chart. Error bars represent mean ± SD (n = 3). ns, no significance, * *p* < 0.05, ** *p* < 0.01. *p*-values were calculated using a Two-way ANOVA using Tukey’s multiple comparison test. dpa: days post-amputation. Figure S6: Domain architectures of BTK proteins. (a) Multiple sequence alignment of BTK across species, with amino acid conservation highlighted as follows: black for identical residues, pink for 75% conservation, and blue for 50% conservation. Key functional sites are boxed: the red box indicates the highly conserved Cys481 residue (the ibrutinib binding site), and the blue box marks the conserved Tyr551 residue (the phosphorylation site). (b) Conserved domain architecture of the BTK protein. Domains were identified using the NCBI Conserved Domain (CD) search tool and visualized with TBtools software. Species abbreviations: HUMAN (*Homo sapiens*), MOUSE (*Mus musculus*), XENLA *(Xenopus laevis*), DANRE (*Danio rerio*), DROME (*Drosophila melanogaster*), and DC (*Dugesia constrictiva*). Figure S7: *BTK* RNAi inhibited the stem cell differentiation into pigment cells in planarians. (a,b) WISH (a) and qRT-PCR (b) analysis of pigmentation genes in intact planarians. Scale bar: 200 µm. *p*-values were shown above the chart. Error bars represent mean ± SD (n = 3). ** *p* < 0.01. (c,d) WISH (c) and qRT-PCR (d) analysis of pigmentation genes in regenerating planarians. *p*-values were shown above the chart. Error bars represent mean ± SD (n = 3). ** *p* < 0.01. dpa: days post-amputation. Table S1: Mortality of planarians exposed to different concentrations of IB for 96 h (n = 20). Table S2: Primers used in this experiment. The Original Images for Blots: Figure S8: Original Images for Figure 6g WB analysis of Cleaved Caspase-3 protein expression in intact planarians following exposure to IB. Figure S9: Original Images for Figure 6h WB analysis of Cleaved Caspase-3 protein expression inregenerating planarians following exposure to IB. Figure S10: Original Images for Figure 9h for Intact. WB analysis of Cleaved Caspase-3 levels in intact planarians after BTK RNAi to assess apoptosis. Figure S11: Original Images for Figure 9h for 10 dpa. WB analysis of Cleaved Caspase-3 levels in regenerating planarians after BTK RNAi to assess apoptosis.

## Abbreviations

The following abbreviations are used in this manuscript:

IB	Ibrutinib
BTK	Bruton’s Tyrosine Kinase
qRT-PCR	Quantitative Reverse Transcription Pcr
WISH	Whole-Mount in situ Hybridization
RNAi	Rna Interference
ROS	Reactive Oxygen Species
CLL	Chronic Lymphocytic Leukemia
MCL	Mantle Cell Lymphoma
DMSO	Dimethylsulfoxide
LC_50_	Median Lethal Concentration
DCFH-DA	2′, 7′-Dichlorofluorescein Diacetate
LSD	Least Significant Difference
CNS	Central Nervous System
H3P	Histone H3 Phosphorylated at Serine 10
