# Supplementary material for: Developmental Toxicity of Ibrutinib: Insights from Stem Cell Dynamics and Neural Regeneration in Planarians
Source: Biomolecules. 2025 Nov 29;15(12):1665. doi: 10.3390/biom15121665 (PMC12731123; doi:10.3390/biom15121665)
Supplement: Supplementary file 1 [file biomolecules-15-01665-s001.zip › biomolecules-3928672-supplementary r1.pdf]

Supporting Information for
Developmental toxicity of Ibrutinib: insights from stem cell dynamics and neural regeneration in planarians

Weiyun Guo[1, 2] · Baijie Jin[1]·Nannan Li[1]·Dandan Sun[1]·Dezeng Liu[1]·Zimei Dong[1*]·Guangwen Chen[1*]

1. *College of Life Science, Henan Normal University, Xinxiang 453007, China*

2. *School of Life Sciences and Technology, Henan Medical University, Xinxiang 453003, China*

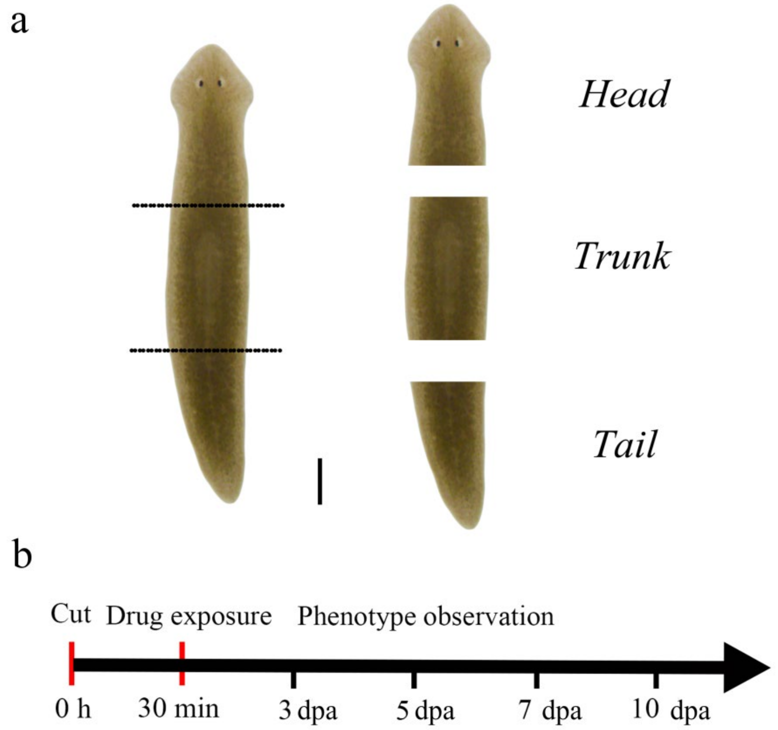

Figure S1. Schedule of the experimental procedure. (**a**) Schematic illustration of planarian amputation. Dashed line: transverse amputation site. Scale bar: 500 μm. (**b**) Schematic overview of the drug exposure protocol in regenerating planarians. dpa: days post-amputation.

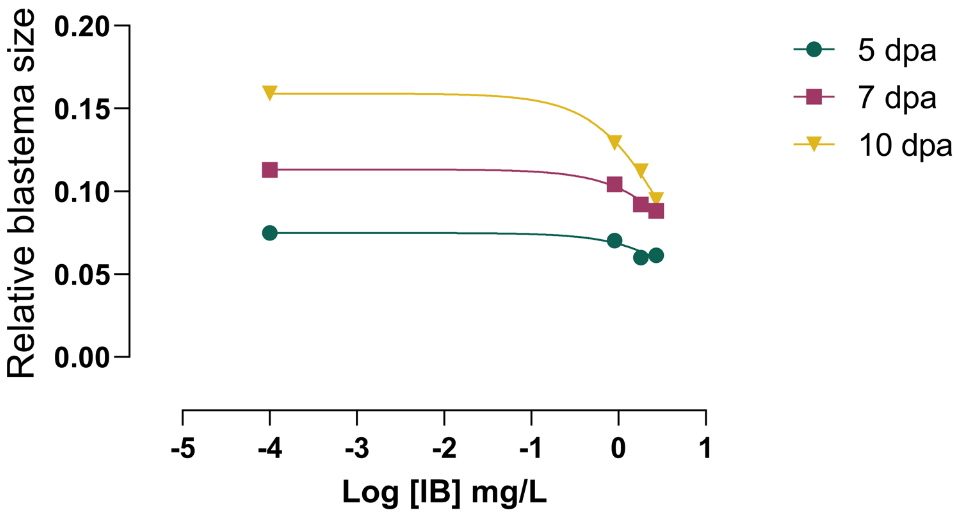

Figure S2 Dose-response curve of IB inhibition on blastema size.

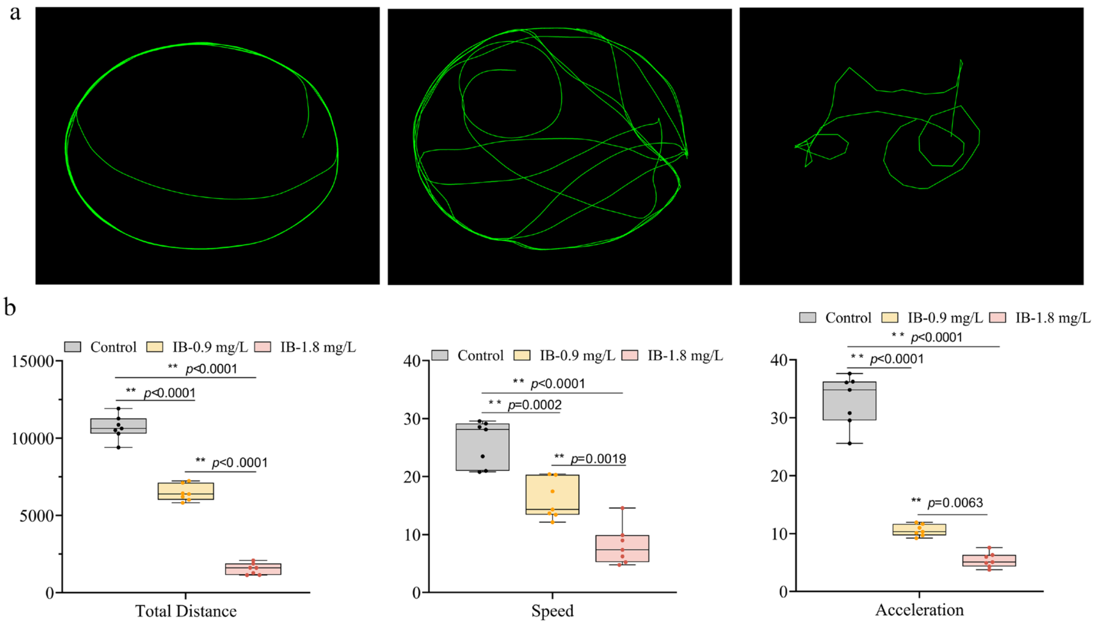

Figure S3 IB exposure changed locomotor behavior in planarians. (**a**) Movement trajectories. (b) Analysis of total movement distance, average speed and average acceleration. The units of distance, speed and acceleration was mm, mm s^{-1}, and mm s^{-2}, respectively. p-values were shown above the chart. Error bars represent mean ± SD (n = 3). $^{**}p < 0.01$. p-values were calculated using a One-way ANOVA with Tukey's multiple comparison test.

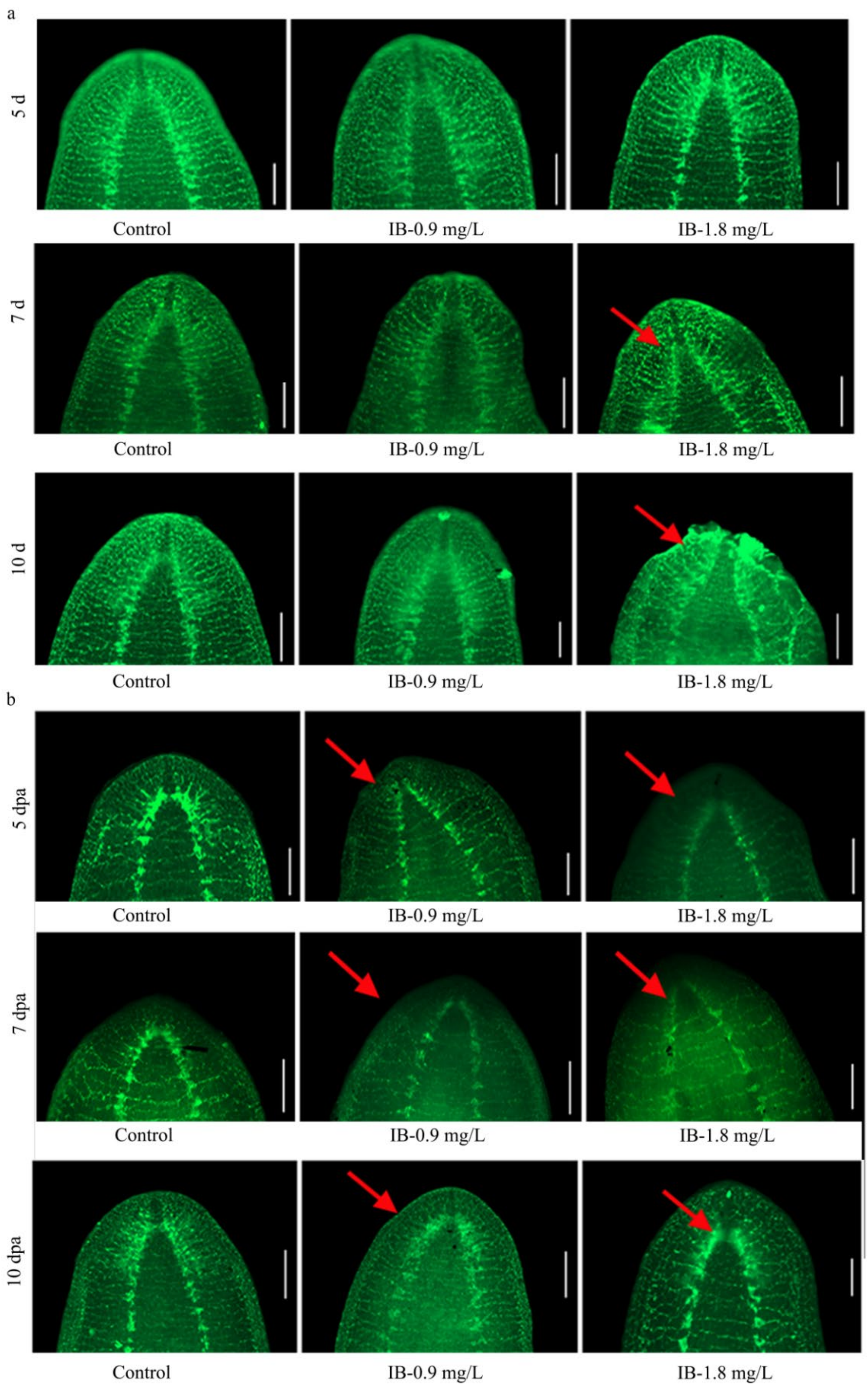

Figure S4. A higher magnification view of neural structural abnormalities in intact (a) and regenerating (b) planarians exposed to IB. Arrow: structural abnormalities. Scale bar: 200 μm. dpa: days post-amputation.

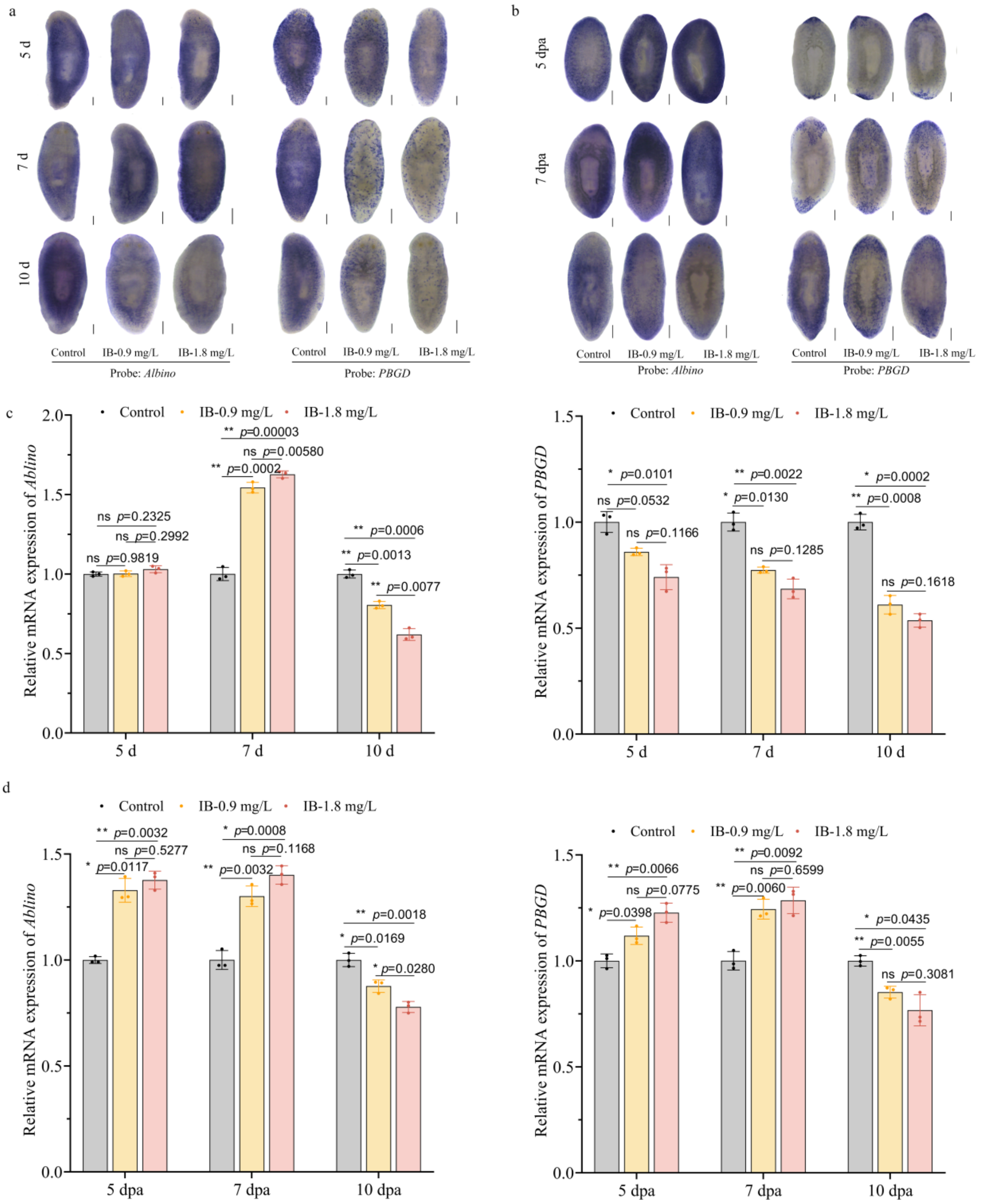

Figure S5 IB exposure disturbed the stem cell differentiation into pigment cells in planarians. (**a,b**) WISH analysis of stem cell differentiation into pigment cells in intact (a) and regenerating (b) planarians. Scale bar: 200 μm. n=8. **(c,d)** qRT-PCR analysis of stem cell differentiation into pigment cells in intact (c) and regenerating (d) planarians. *p*-values were shown above the chart. Error bars represent mean ± SD (n = 3). ns, no significance, **p* < 0.05, ***p* < 0.01. *p*-values were calculated using a Two-way ANOVA using Tukey's multiple comparison test. dpa: days post-amputation.

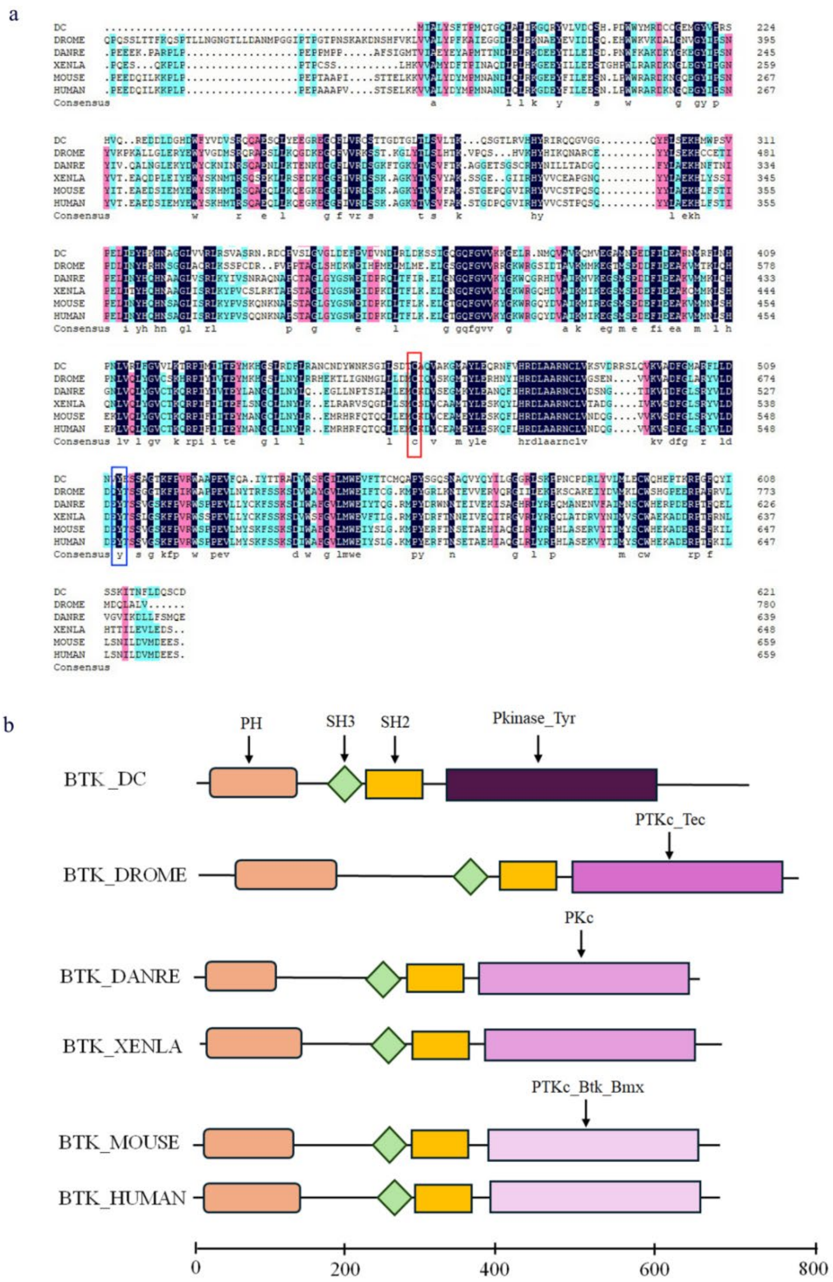

Figure S6 Domain architectures of BTK proteins. (a) Multiple sequence alignment of BTK across species, with amino acid conservation highlighted as follows: black for identical residues, pink for 75% conservation, and blue for 50% conservation. Key functional sites are boxed: the red box indicates the highly conserved Cys481 residue (the ibrutinib binding site), and the blue box marks the conserved Tyr551 residue (the phosphorylation site). (b) Conserved domain architecture of the BTK protein. Domains were identified using the NCBI Conserved Domain (CD) search tool and visualized with TBtools software. Species abbreviations: HUMAN (*Homo sapiens*), MOUSE (*Mus musculus*), XENLA (*Xenopus laevis*), DANRE (*Danio rerio*), DROME (*Drosophila melanogaster*), and DC (*Dugesia constrictiva*).

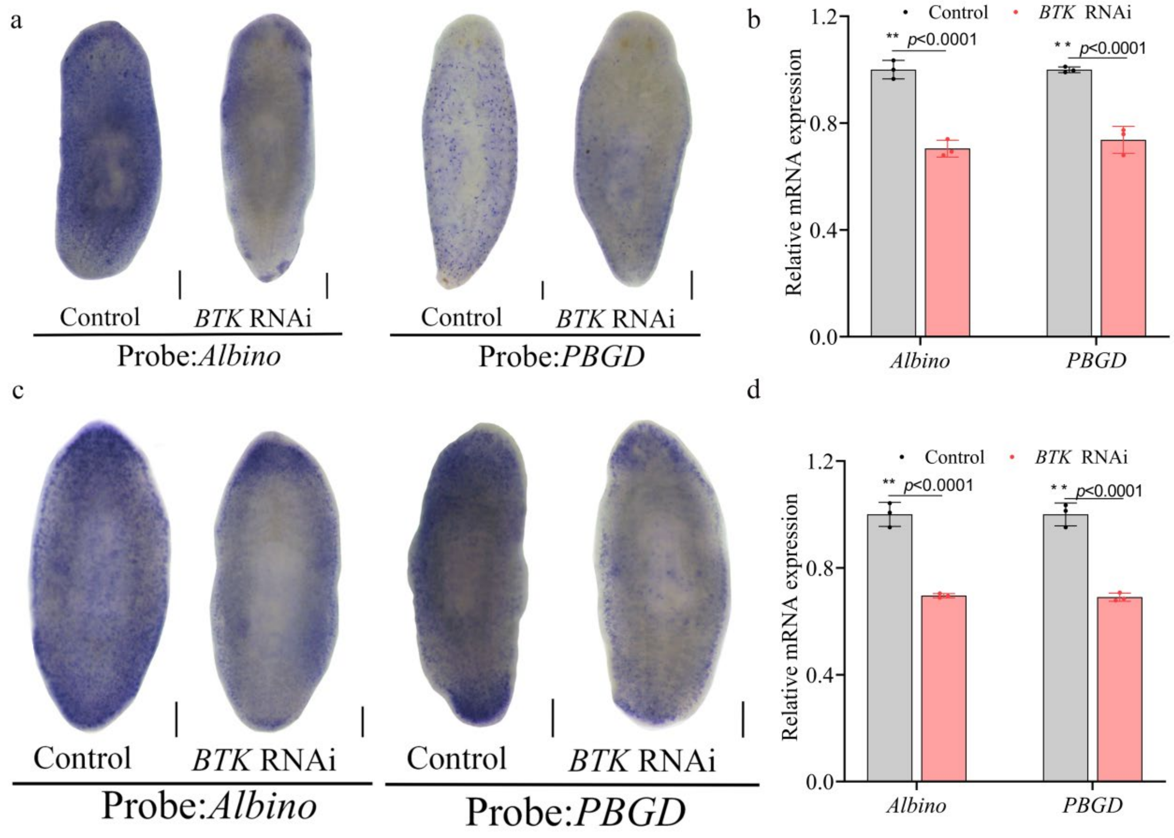

Figure S7 *BTK* RNAi inhibited the stem cell differentiation into pigment cells in planarians. (**a-b**) WISH (**a**) and qRT-PCR (**b**) analysis of pigmentation genes in intact planarians. Scale bar: 200 μm. *p*-values were shown above the chart. Error bars represent mean ± SD (n = 3). **p* < 0.05, ***p* < 0.01. (**c-d**) WISH (**c**) and qRT-PCR (**d**) analysis of pigmentation genes in regenerating planarians. *p*-values were shown above the chart. Error bars represent mean ± SD (n = 3). ***p* < 0.01. dpa: days post-amputation.

Table S1 Mortality of planarians exposed to different concentrations of IB for 96 h (n = 20).

Concentration of IB		1 mg/L	2 mg/L	3 mg/L	4 mg/L	5 mg/L	6 mg/L
mortality	Control	0	0	0	0	0	0
	Group1	2	0	6	12	20	20
	Group2	0	0	4	10	20	20
	Group3	0	2	6	10	20	20

Table S2 Primers used in this experiment

gene	primer	sequence (5′-3′)
ef2	RT-F	GAGTTGCGGTCATGTAGAGTCTGT
	RT-R	AAGCGATCCTGTTGTGTCCTACC
piwiA	RT-F	GATGGTGTAGGAGAT1TCGCAACT
	RT-R	CGGAGAT1GCAGTGCCTTTAGTAGT
	Probe-F	AAGAGAGAT1AGGAAGACTGCG
	Probe-R	GATCACTAATACGACTCACTATAGGGGAGAT1GCTGTGCCTTTAGTAGT
prog1	RT-F	GATGGTGTAGGAGAT1TCGCAACT
	RT-R	CGGAGAT1GCAGTGCCTTTAGTAGT
	Probe-F	CTGGTAAAGAAAGTGAATCTGAAGGT
	Probe-R	GATCACTAATACGACTCACTATAGGGTCCTTTTTCACACTGCTCTACTTTTT
AGAT1	RT-F	GTCCAGAAAGACCCTGCTTACAAAT
	RT-R	CAATGACAACCACTGTGAACTGATG
	Probe-F	TGTTTGGGCATGGAATG
	Probe-R	GATCACTAATACGACTCACTATAGGGCGAATCTTCATCGGCAC
prss12	RT-F	ACAATGCACCGTCATATTCAACCTT
	RT-R	GACATCAGCAGCACTGTAATCCAT
	Probe-F	TGAAGGCACTACACAGTTCTCT
	Probe-R	GATCACTAATACGACTCACTATAGGGGTAATCACCAGTAACAGCGGAAT
Albino	RT-F	ATGTCATCTCCGAAAACCAGAG
	RT-R	GTTAGCGGTGTGATTGTGCC
PBGD	RT-F	GAGACCTGCGATGATGCTGTAG
	RT-R	AATTGAGCAGTTCTACGAACCG
PC2	RT-F	GATTGCTGGATTGCGTATGTTAG
	RT-R	TGGACAGCGGATGGTCAGTAT
Opsin	Probe-F	GTTTTAGGAAATCTTCTCGTGCTT
	Probe-R	GATCACTAATACGACTCACTATAGGGACGCCTATTTCAGTGCTCATT

caspase3	RT-F	TTTATGGCTTTGAGGATTCTGTC
	RT-R	CCGAGTCACATTTATCCCCTTT
BTK1	RT-F	AATCGTCGTTCAATGGGTTGTCAAG
	RT-R	GATTGGCTAAGGCTGCTGCTACA
	RNAi-F	GGGGTACCTAGCAGCAGCCTTAGCCAAT
	RNAi-R	GCTCTAGAACAACCTTCTCGATTCTCCTCTA
	Probe-F	TAGCAGCAGCCTTAGCCAAT
	Probe-R	GATCACTAATACGACTCACTATAGGGACAACCTTCTCGATTCTCCT CTA
BTK2	RT-F	ATGATGCGTCGAGTAGTACCAGAG
	RT-R	TGTCGTCCGCCAAGTATCCAAT
	RNAi-F	GGGGTACCGTGAAGAACGAGACGATTGGAT
	RNAi-R	GCTCTAGACAACAAGACCGCCAGCATT
	Probe-F	GTGAAGAACGAGACGATTGGAT
	Probe-R	GATCACTAATACGACTCACTATAGGGCAACAAGACCGCCAGCATT
