# Supplementary material for: Developmental Toxicity of Ibrutinib: Insights from Stem Cell Dynamics and Neural Regeneration in Planarians
Source: Biomolecules. 2025 Nov 29;15(12):1665. doi: 10.3390/biom15121665 (PMC12731123; doi:10.3390/biom15121665)
Supplement: Supplementary file 1 [file biomolecules-15-01665-s001.zip › Original Images for Blots.pdf]

Supporting Information of Electrophoretic gels and blots for Developmental toxicity of Ibrutinib: insights from stem cell dynamics and neural regeneration in planarians

Weiyun Guo[1, 2] · Baijie Jin[1]·Nannan Li[1]·Dandan Sun[1]·Dezeng Liu[1]·Zimei Dong[1*]·Guangwen Chen[1*]

1. College of Life Science, Henan Normal University, Xinxiang 453007, China

2. School of Life Sciences and Technology, Henan Medical University, Xinxiang 453003, China

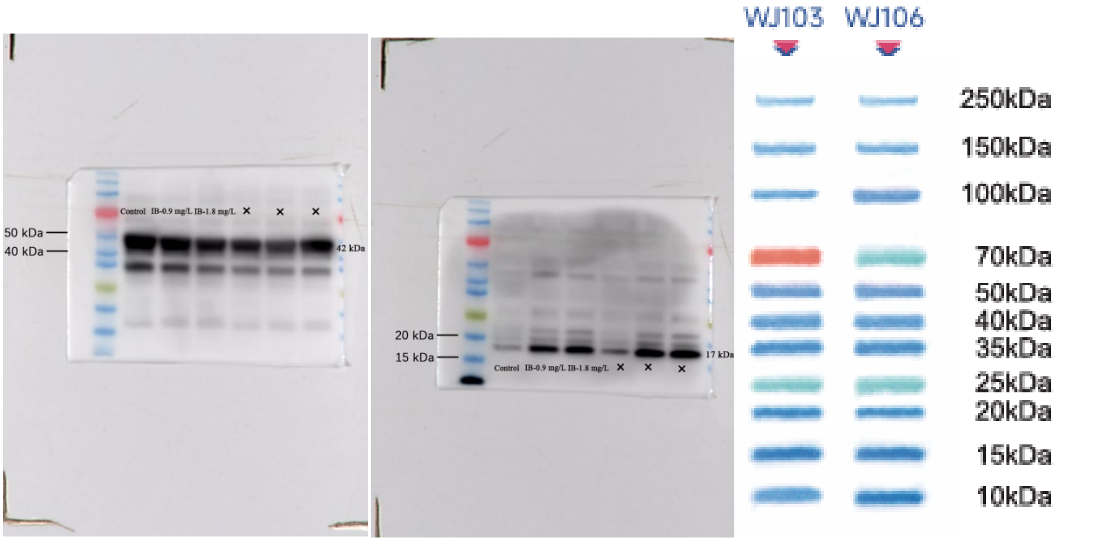

Figure S8: Original Images for Figure 6g WB analysis of Cleaved Caspase-3 protein expression in intact planarians following exposure to IB.

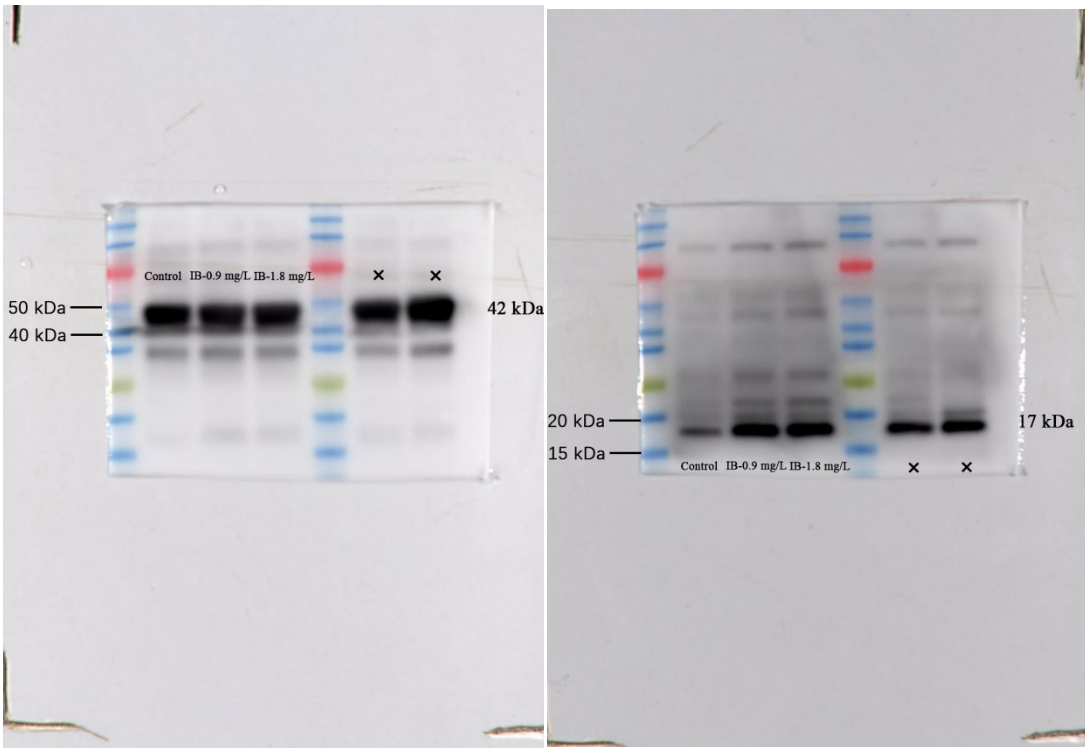

Figure S9: Original Images for Figure 6h WB analysis of Cleaved Caspase-3 protein expression inregenerating planarians following exposure to IB.

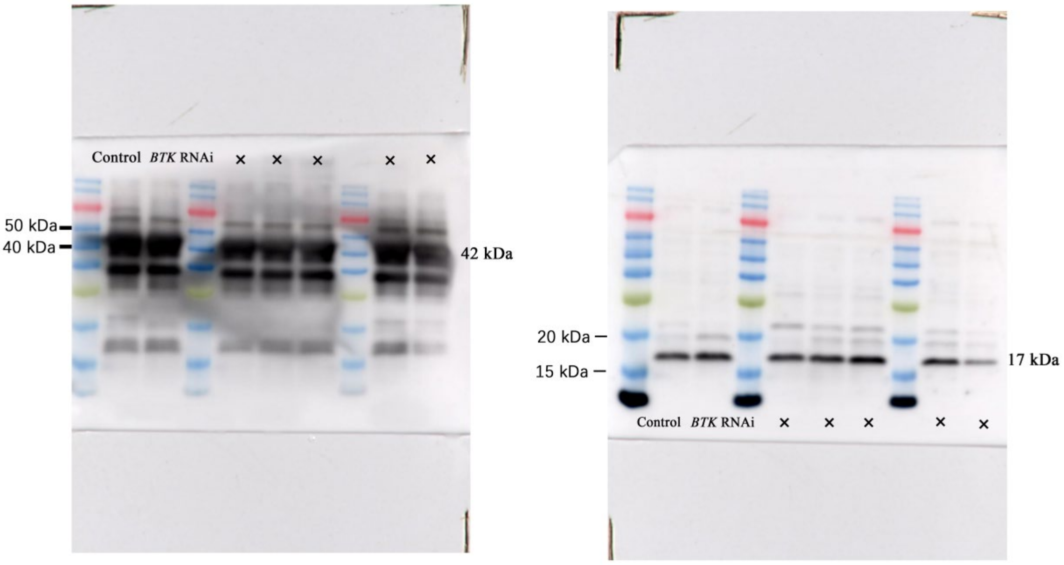

Figure S10: Original Images for Figure 9h for Intact. WB analysis of Cleaved Caspase-3 levels in intact planarians after *BTK* RNAi to assess apoptosis.

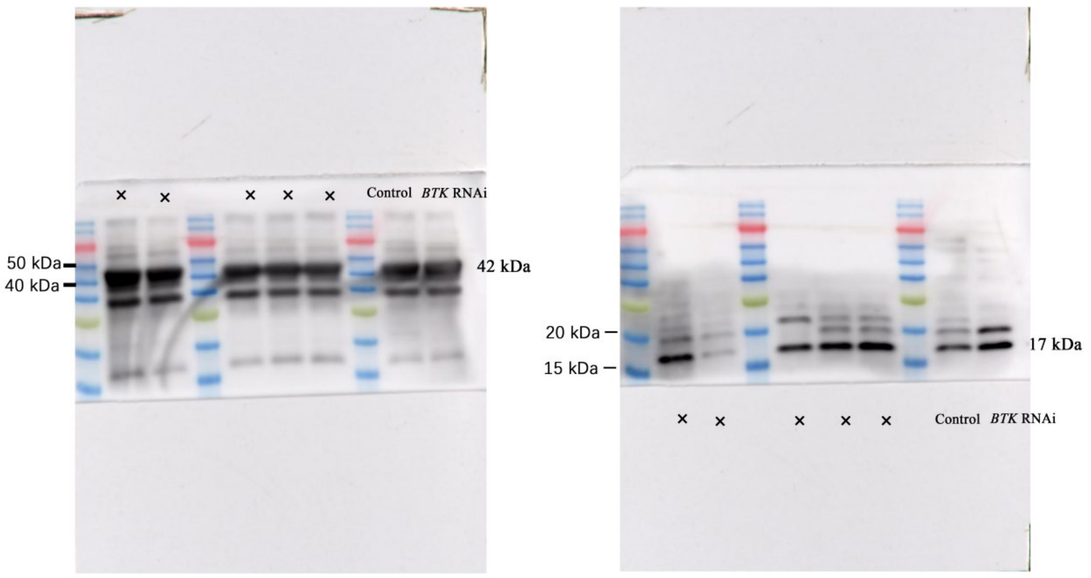

Figure S11: Original Images for Figure 9h for 10 dpa. WB analysis of Cleaved Caspase-3 levels in regenerating planarians after *BTK* RNAi to assess apoptosis.
